# Functional conservation of sequence determinants at rapidly evolving regulatory regions across mammals

**DOI:** 10.1371/journal.pcbi.1006451

**Published:** 2018-10-05

**Authors:** Iksoo Huh, Isabel Mendizabal, Taesung Park, Soojin V. Yi

**Affiliations:** 1 School of Biological Sciences, Institute of Bioengineering and Bioscience, Georgia Institute of Technology, Atlanta, GA, United States of America; 2 College of Nursing, The Research Institute of Nursing Science, Seoul National University, Seoul, Korea; 3 Department of Statistics, College of Natural Sciences, Seoul National University, Seoul, Korea; Ottawa University, CANADA

## Abstract

Recent advances in epigenomics have made it possible to map genome-wide regulatory regions using empirical methods. Subsequent comparative epigenomic studies have revealed that regulatory regions diverge rapidly between genome of different species, and that the divergence is more pronounced in enhancers than in promoters. To understand genomic changes underlying these patterns, we investigated if we can identify specific sequence fragments that are over-enriched in regulatory regions, thus potentially contributing to regulatory functions of such regions. Here we report numerous sequence fragments that are statistically over-enriched in enhancers and promoters of different mammals (which we refer to as ‘sequence determinants’). Interestingly, the degree of statistical enrichment, which presumably is associated with the degree of regulatory impacts of the specific sequence determinant, was significantly higher for promoter sequence determinants than enhancer sequence determinants. We further used a machine learning method to construct prediction models using sequence determinants. Remarkably, prediction models constructed from one species could be used to predict regulatory regions of other species with high accuracy. This observation indicates that even though the precise locations of regulatory regions diverge rapidly during evolution, the functional potential of sequence determinants underlying regulatory sequences may be conserved between species.

## Author summary

Regions of the genome that do not encode genes but affect expression of other genes, such as enhancers and promoters, are referred to as regulatory regions. Because of their regulatory functions, it was thought that enhancers and promoters should be evolutionarily conserved. Regulatory regions can be now epigenomically identified because they are marked by specific modifications of histone tails at the chromatin level. Interestingly, when we compare epigenomically identified regulatory regions from different mammals, the specific positions of regulatory regions are often divergent between species. Enhancers in particular are highly divergent between species. In this study, we show that we can find sequence fragments that are statistically enriched in enhancers and promoters of different species, and that the degree of statistical enrichment can explain different levels of evolutionary sequence conservation between enhancers and promoters. We further constructed predictive models of enhancers and promoters using the enriched sequence fragments, and show that these models can not only accurately predict enhancers and promoters of the same species, but works comparably well when applied to other species. These results indicate that even though the specific positions of regulatory regions have diverged between species, the functions of sequence fragments that comprise those regions may be conserved.

## Introduction

Epigenomic modifications such as histone modifications and DNA methylation play critical roles in development, regulation, and diseases. The study of epigenetic modifications has made great strides in recent decades, and the specific combinations of different epigenome components in distinct biological conditions are rapidly being discovered [1]. In particular, epigenomic profiling is widely used to empirically identify regulatory regions including enhancers and promoters using chromatin immunoprecipitation with massively parallel DNA sequencing (ChIP-seq). For example, genomic regions enriched for histone H3 lysine 27 acetylation (H3K27ac) are considered as active enhancers [2, 3]. On the other hand, enrichment of histone H3 lysine 4 trimethylation (H3K4me3), in particular together with H3K27ac, indicates active promoters [4–6].

Beyond identifying regulatory regions, the next challenge is deciphering what factors determine and affect epigenomes. Among potential factors, the importance of *cis*-regulatory sequences on the epigenome is well appreciated. Several *cis*-regulatory sequences based predictive models have been constructed to classify regulatory regions [7–10]. For example, a recent study reported random forest classifier models from the human genome that could predict regulatory regions marked by H3K27ac and H3K4me3 modifications with relatively high accuracy [11].

Even though our understanding of the true nature of the relationship between specific histone modifications and regulatory regions is sure to undergo much more revisions, these technical advances in genome-wide epigenomic profiling brought new approaches to study evolution of regulatory regions. Instead of having to rely on experimentally characterized comparative transcription factor binding assays [12–14] and/or regions that retain sequence similarities [15–18], enhancers and promoters can be identified based on the distribution of specific epigenomic modifications such as H3K4me3 and H3K27Ac across different species [6, 19]. Interestingly, these studies show that at the genome-scale, chromosomal locations of enhancers are highly divergent between species [6, 20, 21]. Promoters are also found in divergent locations, although their positions are more constrained than enhancers, since promoters are typically adjacent to transcription units (e.g. [6]). Thus, while regulatory regions can be reliably predicted from sequences within specific genomes [7–11], the precise locations of regulatory regions, in particular of enhancers, diverge rapidly during evolution [6, 18, 20, 21].

It is not necessarily straightforward to reconcile these two aspects of regulatory regions. In the simplest scenario, functional regions such as enhancers and promoters should be evolutionarily conserved since they are subject to purifying selection. Indeed, this idea has been successfully used to identify non-coding sequences with regulatory functions [16, 17, 22, 23]. However, at the genome-scale, regulatory regions harbor little sequence similarities and their locations are highly divergent. Rapid turnover of transcription binding sites [12, 24, 25] and transcription rewiring [26–28] can explain some aspects of regulatory sequence evolution, but many questions still remain [29, 30].

Here, utilizing the wealth of comparative data on epigenomically determined enhancers and promoters, we investigated whether we could identify specific sequence fragments that constitute enhancers and promoters, and if so, whether such sequence fragments were evolutionarily conserved between species. We first performed an exhaustive search to identify sequence fragments that are statistically over-represented in experimentally identified enhancers and promoters of several mammals [6]. A unique aspect of our study is that we focused on distinguishing regulatory regions from nearby regions. Genomic sequences of mammals such as humans are highly heterogeneous in many aspects such as GC contents, transposable element contents, genic contents, and other aspects [31, 32]. By comparing regulatory regions to their nearby non-regulatory regions, we identified sequence fragments that distinguished regulatory regions from its local genomic backgrounds. Our comprehensive exhaustive search revealed numerous sequence fragments that were significantly enriched in regulatory regions compared to nearby regions. Due to the nature of the exhaustive search, some of the identified sequence fragments may be inter-related. To overcome this limitation and identify a subset of sequence fragments that are statistically independent, and to construct prediction models to test evolutionary hypotheses, we employed a machine learning method. Specifically, we used the least absolute shrinkage and selection operator (LASSO) method [33], which can effectively select one variable among the set of highly correlated variables [34]. The LASSO method is also excellent at prediction accuracy [11, 35].

From these procedures, we discovered numerous sequence fragments that are statistically enriched in experimentally verified regulatory regions (referred to as ‘sequence determinants’ henceforth). Intriguingly, sequence determinants obtained from enhancers and promoters show remarkable differences with respect to their impact on functional regions. Moreover, even though sequence determinants themselves exhibit only moderate overlaps between species, prediction models constructed using sequence determinants from different species could be inter-changed to perform as well as prediction models from the focal species. We discuss potential implications of these findings.

## Materials and methods

### Enhancer and promoter data

We used experimental annotations of liver enhancers and promoters from a previous study [6]. Following the definition in this study [6], we considered enhancers to be regions marked only with the H3K27ac mark and promoters to be regions marked with H3K4me3 (with or without H3K27ac). We selected data from seven ‘high-quality’ mammalian genomes as indicated in [6], including *Home sapiens* (human), *Macaca mulatta* (macaque), *Bos taurus* (cow), *Sus scrofa* (pig), *Canis familiaris* (dog), *Rattus norvegicus* [32], and *Mus musculus* (mouse). Each enhancer or promoter was designated as foreground, and a segment of the same length 100,000 base-pairs (100kb) apart from the foreground was selected as the background. We used these ‘regional’ backgrounds to control for potential chromosome effect and/or regional effects. The distance of 100kb between the foreground and background was selected since several genomic features such as linkage disequilibrium blocks and GC contents show correlations that extend to ~ 100kb [32, 36]. We obtained the genome sequences using the R Bioconductor libraries “BSgenome” [37]. Backgrounds that had greater than 50% of nucleotides missing (not sequenced) were discarded (Table 1), and put information on overlapped proportions between foreground and background in S1 Table.

**Table 1:** Summary of the datasets used in this study.

	Enhancers				Promoters			
	Total		Conserved		Total		Conserved	
	Foreground (Background)^1^	Mean (SD) of lengths^2^	Foreground (proportion)	Mean (SD) of lengths^2^	Foreground (Background)^1^	Mean (SD) of lengths^2^	Foreground (proportion)	Mean (SD) of lengths^2^
Human	29137 (29007)	3275 (2551)	305 (1.0%)	7531 (5741)	12035 (11981)	2497 (922)	2039 (16.9%)	2772 (991)
Macaque	22089 (21732)	2514 (1791)	379 (1.7%)	3957 (3191)	11162 (10472)	2102 (789)	2085 (18.7%)	2271 (825)
Cow	31971 (31884)	1988 (1519)	457 (1.4%)	3175 (2563)	13792 (13766)	2385 (876)	2103 (15.2%)	2689 (954)
Pig	23804 (21229)	3322 (2432)	349 (1.5%)	6720 (5611)	11114 (9823)	2046 (909)	2086 (18.8%)	2368 (1006)
Dog	20070 (20026)	3181 (2212)	324 (1.6%)	5265 (3716)	11093 (11055)	2401 (903)	2103 (19.0%)	2574 (975)
Rat	22416 (21642)	2792 (2250)	384 (1.7%)	4656 (4539)	17086 (16389)	1765 (1030)	2154 (12.6%)	2296 (1139)
Mouse	18396 (18339)	2572 (1927)	355 (1.9%)	4148 (5186)	15164 (15104)	2648 (1221)	2042 (13.5%)	3150 (1345)

^1^ If some of background regions were discarded because they had >50% N/A nucleotides.

^2^ Statistic using foreground data set.

### Enhancer and promoters in orthologous locations

Those enhancers and promoters found in orthologous locations across species were identified as conserved (Table 1). Specifically, for each human enhancer or promoter we retrieved the 17 eutherian EPO multiple alignment using Ensembl REST API [38] and determined if the region was conserved or not based on whether all other 6 species also showed the same histone mark(s) in the orthologous region. For species with different genome assemblies in the alignment, we converted the coordinates using Ensembl assembly converter [39].

### Exhaustive search for sequence determinants

**Fig 1:**
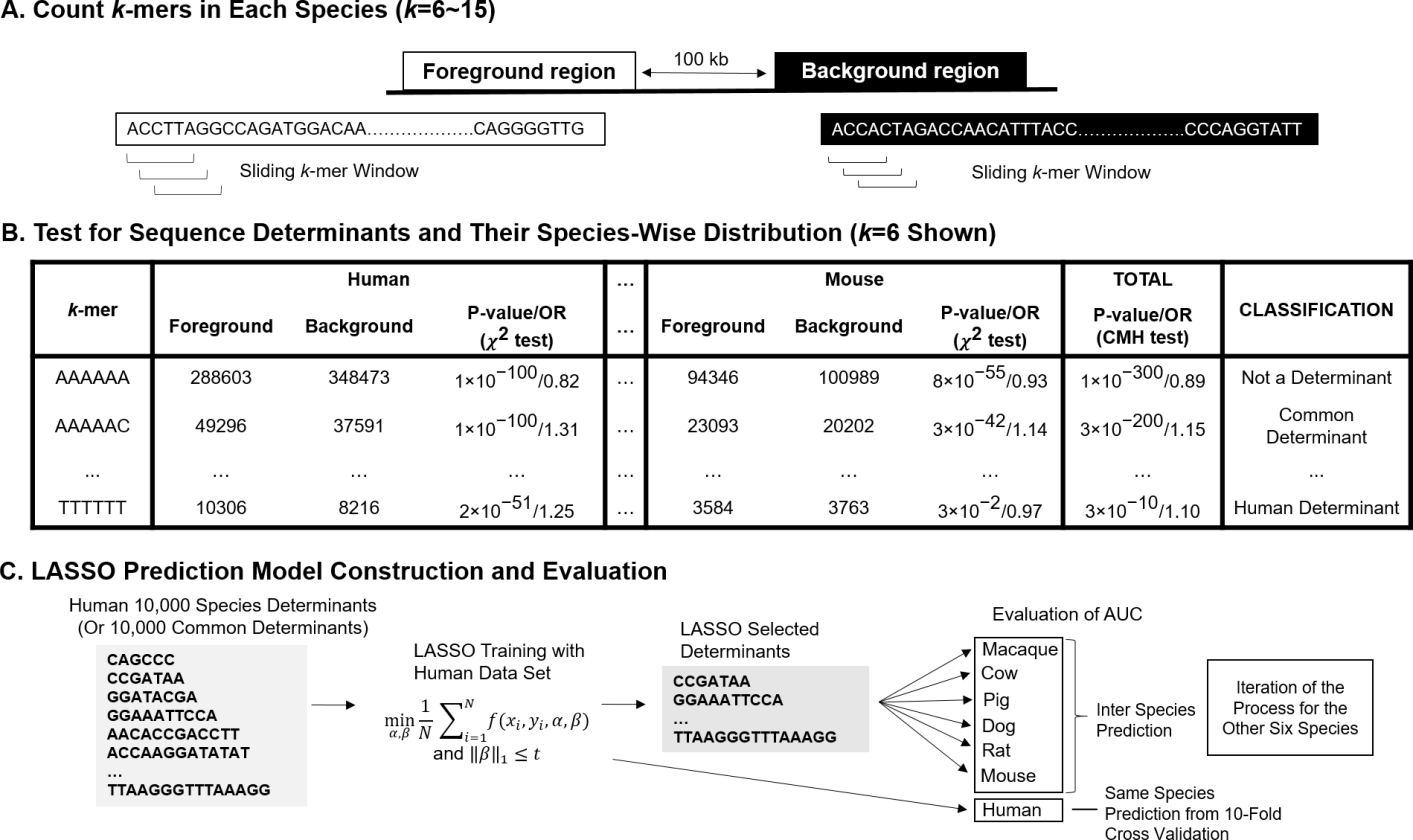
Overall workflow for the exhaustive search and construction of prediction models. (A) Each sequence fragment is counted and summarized in a k-mer sliding window for foreground (regulatory regions) and background (control). (B) Using the counts from the sliding window, a χ^2^ test for each sequence fragment (of each species) is conducted to determine whether that sequence fragment is significantly over-represented in the foreground compared to the background. Significantly over-represented sequence fragments are named ‘species sequence determinants’. In addition, the CMH test [34] is used to detect sequence determinants that are present in all seven species (‘common sequence determinants’). (C) The least absolute shrinkage and selection operator (LASSO) prediction models are constructed for each species using a subset of sequence determinants from (B). These sequence determinants are randomly selected within the stratification of GC content and fragment length. After LASSO selection, which removes some of redundant or non-significant determinants, the resulting models were applied to its own species (same-species prediction), or the other six species (inter-species prediction) to evaluate prediction performances.

We examined whether specific sequence fragments in the foreground were over-represented compared to the backgrounds by statistical testing. We used sliding windows with a specific length (from 6-mers to 15-mers), moving from the 5’ end to the 3’ end in each foreground or background (Fig 1). As the window moved by a base-pair (bp), a sequence fragment within that bin was captured and recorded. Following this sliding window analysis, counts of each sequence fragment in the foreground and background were obtained. For each sequence fragment, we constructed a 2×2 contingency table that contained counts of a sequence determinant in each of foreground and background region (Table 2), and we used the odds ratio (OR) as a measure of over-representation in foreground, compared to background. The magnitude of OR indicated how strongly over-enriched a specific element was in regulatory regions, which we also referred to as ‘effect size’ in this study.

**Table 2:** 2×2 contingency table to apply χ^2^ test to identify significant sequence determinants.

	Count of a target sequence fragment^1^	Count of the other sequence fragments^1^	Total count
Foreground	N_11_ (N_1+_× N_+1_/N_++_)	N_12_ (N_1+_× N_+2_/N_++_)	N_1+_
Background	N_21_ (N_2+_× N_+1_/N_++_)	N_22_ (N_2+_× N_+2_/N_++_)	N_2+_
Total count	N_+1_	N_+2_	N_++_

^1^Numbers in the parenthesis are expected numbers to calculate the χ^2^ test statistics. For example, for a sequence fragment (e.g.”AACCGGTT”), N_11_ is its observed count in the foreground regions, N_12_ is the observed count of sequence fragments that are not “AACCGGTT”, but has the same length in the foreground regions, and N_21_ and N_22_ are the counterpart of the N_11_ and N_12_ in the background regions, respectively. The sign “+” means row-wise sums (N_1+_, N_2+_), column-wise sums (N_+1_, N_+2_), or total sum (N_++_). OR is estimated as N_11_×N_22_/(N_12_×N_21_).

We used the χ^2^ test to test the following null and alternative hypotheses:

$$H_{0}:\;OR=1,$$ (1)

$$H_{1}:\;not\;H_{0.}$$ (2)

If the expected count of a sequence fragment in any of the cell in the 2×2 contingency table was lower than 5, we used the Fisher’s exact test instead. The resulting *P*-values were corrected for multiple testing using the false discovery rate (FDR) approach [40]. Following these procedures, a ‘sequence determinant’ in the statistical sense was identified as a sequence fragment whose FDR *Q*-value was equal to or less than 0.05 and the OR was greater than 1. In the process, we tested only sequence determinants that appeared over 100 times to avoid selecting rare sequence determinants of negligible biological relevance. For example, for 15-mers in the human enhancer data set, most sequence fragments (63 million out of 70 million) occurred only once. We repeated this procedure for each of the seven species and identified ‘species sequence determinants’.

### Common sequence determinants

We identified ‘common sequence determinants’ as sequence fragments that are enriched in foreground regions compared to the background regions across the seven mammalian species. For the purpose, we used the Cochran-Mantel-Haenzel (CMH) test [41] to identify enrichment of sequence determinants from multiple data sets using a conditional variable, which is a nominal covariate such as the species index [41, 42]. The CMH test is also equivalent to the score type test of logistic regression, which has advantages in the handling of sparse count data sets [42]. Consequently, we used the CMH to test the null hypothesis,

$$H_{0}{:\;\mathrm{OR}}_{|\mathrm{species}}{=1,\mathrm{where}\;\mathrm{OR}}_{|\mathrm{species}}\;\mathrm{is}\;\mathrm{the}\;\mathrm{conditional}\;\mathrm{OR}\;\mathrm{in}\;\mathrm{presence}\;\mathrm{of}\;\mathrm{the}\;\mathrm{species}\;\mathrm{index}.$$ (3)

$$H_{1}:\;not\;H_{0.}$$ (4)

Common sequence determinants were then defined as those whose OR_|species_>1 for all species and FDR *Q*-value from CMH ≤ 0.05.

### LASSO prediction models using sequence determinants from exhaustive search

We constructed prediction models that yield predictive scores for each region. We used the least absolute shrinkage and selection operator (LASSO) method [33], which excels at prediction accuracy as well as covariate selection [11, 35]. In the LASSO model, each foreground or background region was regarded as a binary observation (foreground = 1, background = 0). The relative frequency of each sequence determinant was regarded as an explanatory variable. Because the space of all significant sequence determinants was extremely large (S2 and S3 Tables), including all determinants in the LASSO model was not computationally feasible. Instead, we selected 10,000 sequence determinants, sampled according to their distribution of GC content and fragment length, to incorporate in the LASSO models using a stratified sampling approach [43]. Specifically, we stratified the whole sequence determinants by the combination of GC content (ten uniform intervals: [0~0.1],…, (0.9~1.0]) and length (ten lengths: 6,…,15bp). Then we selected samples from each of the stratified subsets so that its number out of the 10,000 was proportional to the number of determinants in the specific subset among the total determinants. To train LASSO models and estimate coefficient of each determinant, we used the R function “glmnet” from the package “glmnet” using R 3.4.0.

To construct prediction models, we used both the 10,000 species sequence determinants and the 10,000 common sequence determinants as input variables, so that we can compare the prediction performances of species determinants and common determinants. We performed two types of predictions. First, we performed same-species prediction, which evaluates prediction AUC through a 10-fold cross-validation process [11, 35, 44, 45]. During the 10-fold cross-validation process, an optimal penalty parameter that provides the smallest test AUC is chosen. We regarded the smallest test AUC as same-species prediction AUC. For inter-species prediction, we used the optimal parameter to construct a prediction model from whole data set of a species and applied the model to the other species to calculate inter-species prediction AUCs. Workflow from the exhaustive search to LASSO is depicted in Fig 1. In most prediction results, we provided two types of AUC, the first one is receive operating characteristic AUC (ROC-AUC) for general performance of prediction and the second one is precision-recall AUC (PR-AUC) for robustness of performance regardless of the ratio between numbers of foreground and background [46].

Among several machine-learning methods, we selected LASSO because of its ability to reduce the number of input variables so that those are not redundant and are statistically meaningful. However, other machine learning methods might be useful as well. For example, when many of sequence determinants have strong relationship in terms of correlation, elastic net that can capture more input variables would be useful to improve prediction performances [47].

### Transcription factor binding sites (TFBS) analysis

We examined the presence of transcription factor binding sites (TFBS) in the sequence determinants using TOMTOM [48]. This tool assesses the similarity between individual sequence input and specific TFBS databases and provides *P*-values and *Q*-values adjusted by FDR. Known TFBS compiled in the JASPAR 2014 Core vertebrate database [49], the HOCOMOCOv10_HUMAN and the HOCOMOCOv10_MOUSE [50] were used. We summarized the proportion of significant (*P* <0.05) TFBS hits as ‘TFBS frequency’. For example, each human sequence determinant was compared to the 641 known TFBS in the HOCOMOCOv10_HUMAN database. The number of significant comparisons out of the total 641 comparisons was referred to as ‘TFBS frequency’. Due to the probabilistic nature of TF binding and the fact that sequence determinants might encode partial or full TFBS, TFBS frequency indicates versatility of a sequence determinant that can be a motif for TFBS binding. For instance, the CAGCCC determinant from the human genome yielded 18 of 641 significant hits, thus TFBS frequency of the determinant was 2.8%. We also used–log_10_min(P) instead of TFBS frequency to evaluate the best match between a k-mer and the motifs in the database.

### Analysis of the relationship between biological factors of sequence determinants

Sequence determinants from the exhaustive search as well as from the LASSO prediction models were further analyzed to explore relationships between their effect sizes and several biological factors such as GC content and TFBS binding properties. For this analysis, we used the following linear model;

$$\log_{2}{\left(\mathrm{OR}\right)}_{i}{∼\mathrm{GC}\;\mathrm{content}}_{i}{+\mathrm{TFBS}\;\mathrm{frequency}}_{i}{+\mathrm{GC}\;\mathrm{content}}_{i}{\times \mathrm{TFBS}\;\mathrm{frequency}}_{i}+\varepsilon_{i},$$ (5)

where *i* is the index of each sequence determinant and ε_i_ ~*N*(0,σ^2^). In this model, we log_2_ transformed the OR values to improve normality. We applied the model to enhancer and promoter sequence determinants from common, human, and mouse sets.

## Results

### Promoter sequence determinants are strongly over-represented relative to enhancer sequence determinants

To identify sequence fragments that are significantly enriched in enhancers or promoters compared to nearby background regions (sequence determinants), we first performed an *exhaustive search*. Briefly, we examined sequence fragments of lengths from 6 to 15 bp, using a sliding window approach (Fig 1). We tested statistical over-representation of the specific sequence fragment in the enhancers or promoters compared to their backgrounds using a contingency table test based on their ORs. The *P*-values were adjusted via the false discovery procedure [40] (Materials and Methods).

**Fig 2:**
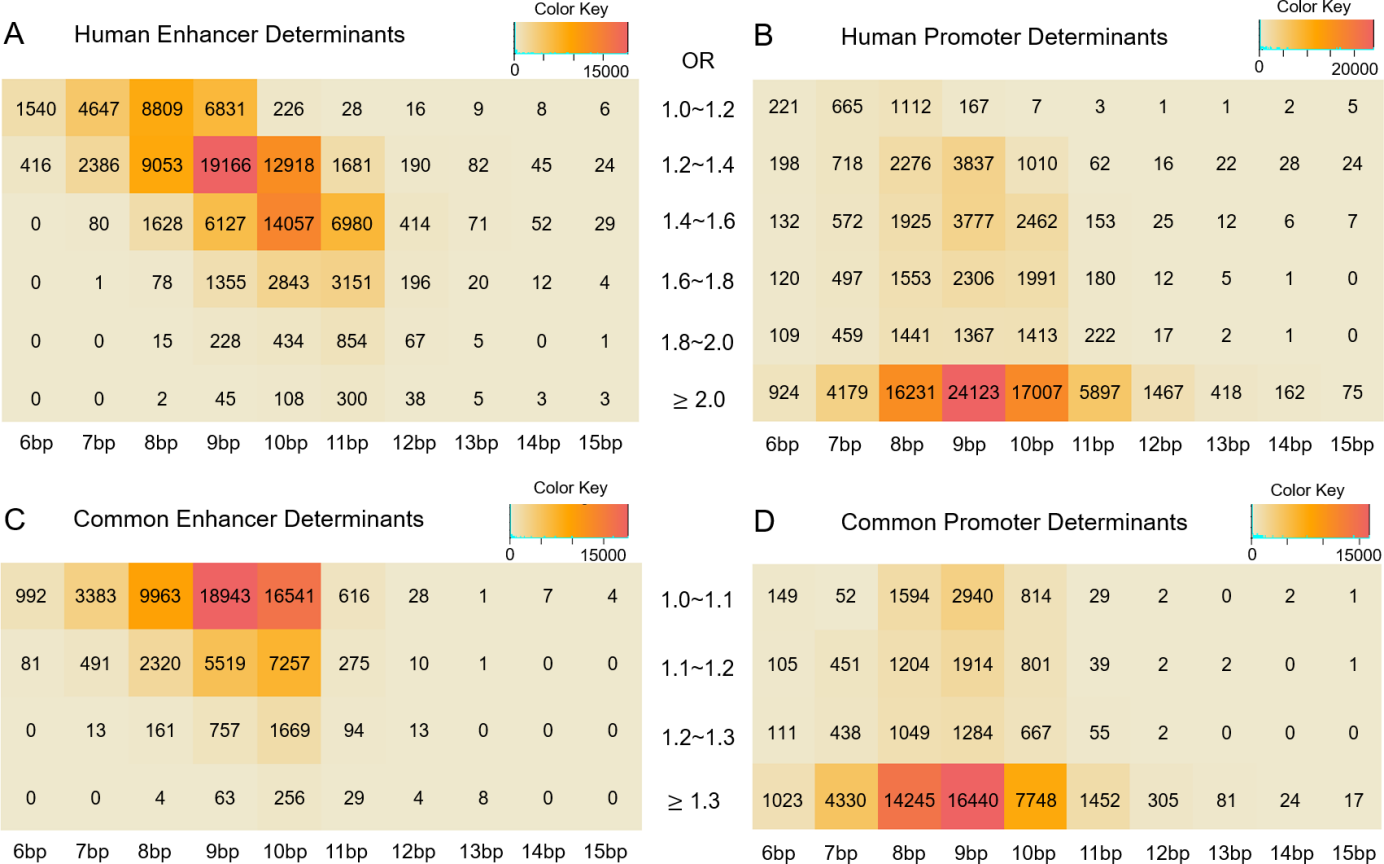
Heatmaps of the sequence determinant counts according to sequence length and OR from the exhaustive search. The X-axis corresponds to the length of sequence determinants and Y-axis to the OR of each sequence determinant. (A) and (B) are from human species sequence determinants while (C) and (D) are from common sequence determinants. The count information of species sequence determinants in the other six species are summarized in S2 and S3 Tables. OR in the heatmaps for the common sequence determinants represents the minimum OR value among all seven individual ORs. The total numbers of sequence determinants are 107,287 in (A), 101,625 in (B), 69,503 in (C), 59,783 in (D).

**Fig 3:**
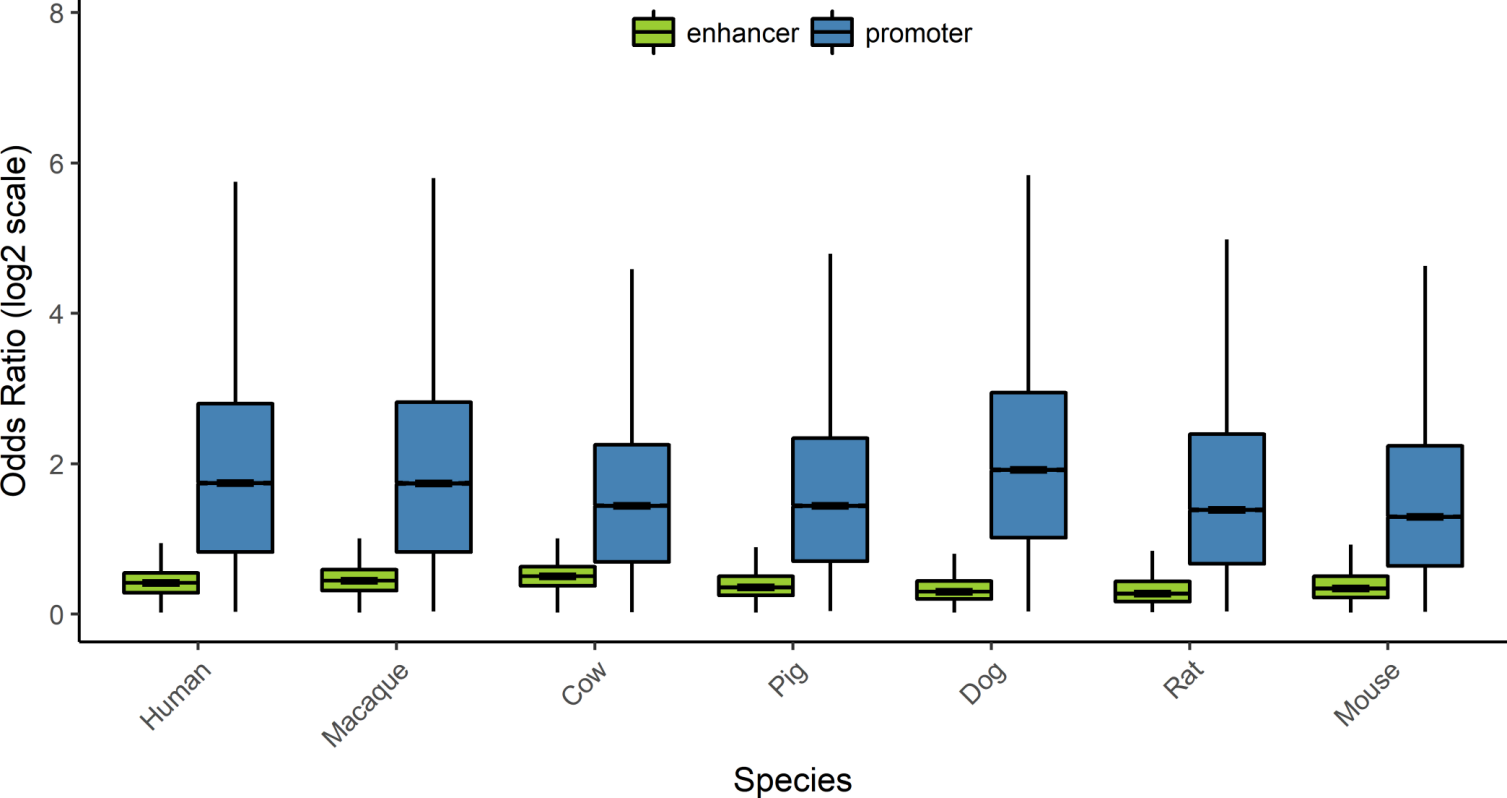
Contrasting odds ratio distributions of enhancer and promoter sequence determinants using boxplots. Sequence determinants from promoters have significantly higher Odds Ratio than those from enhancers in all seven species (*P* < 10^−15^ by Wilcoxon’s rank sum-test in all species).

Following these procedures, we identified numerous sequence determinants associated with enhancers and promoters of each species (referred to as ‘species sequence determinants’, Materials and Methods). Fig 2(A) and 2(B) show the numbers of significant sequence determinants from human enhancers and promoters based on their OR and length. The majority of sequence determinants in enhancers and promoters were found in 7–11 bps. Human enhancer determinants were slightly yet significantly longer than promoter determinants (mean lengths for human enhancers and promoters were 9.20 and 9.01, *P* <1×10^−5^ by two sample *t-*test). However, there was no consistent pattern across the seven mammals when comparing the length of sequence determinants in enhancers and promoters. Sequence determinants were also generally GC-rich and TFBS-rich compared to non-significant sequence fragments (see below). Remarkably, with respect to OR, sequence determinants from enhancers and promoters were highly distinct. Strongly enriched sequence determinants, such as those with OR ≥ 2.0, were 140-fold more abundant in promoters than in enhancers (Fig 2). Accordingly, the ORs of sequence determinants were significantly higher in promoter sequence determinants than in enhancer sequence determinants (*P* < 10^−15^ by Wilcoxon’s rank sum-test in all seven species, Fig 3).

We then examined sequence determinants that occurred more frequently than expected in all seven mammalian species, which we referred to as ‘common sequence determinants’ (Materials and Methods). Similar to the results from the above analysis, common sequence determinants had higher ORs in promoters than in enhancers (*P* < 10^−15^ by Wilcoxon’s rank sum-test, Fig 2(C) and 2(D), S4 Table). When we compared the entries of common sequence determinants to those of species sequence determinants, we found that 39% and 57% of all human enhancer and promoter determinants overlapped with common enhancer and promoter sequence determinants, respectively (S5 Table). Therefore, regardless of their species-wise distribution, sequence determinants that mark promoters tended to have significantly greater OR thus presumably stronger effects on regulatory potential of target regions in terms of marginal effect size, compared to those found in enhancers.

### LASSO approach supports different effect sizes of enhancer and promoter sequence determinants

The exhaustive search allowed us to identify all sequence determinants that were marginally enriched. However, some sequence determinants might be highly correlated with each other, because they were extracted from overlapping regions (Fig 1). The LASSO approach is capable of selecting one variable among the highly correlated variable sets, in addition to selecting variables of substantial effect [33]. Therefore, we next used the LASSO approach to select essential variables among the many correlated variables, and to construct prediction models that discriminate enhancers and promoters from their corresponding background regions (Materials and Methods). The total numbers of sequence determinants from the human enhancers and promoters were 107,287 and 101,625, respectively (S2 and S3 Tables). AUCs increased as the number of input sequence determinants increased, to stabilize around 7,000 sequence determinants (S1 Fig). We thus chose 10,000 sequence determinants for each set of sequence determinants using a stratified sampling approach [43], to select a subset that is representative of the original distribution with respect to GC contents and lengths (Materials and Methods). Following these steps, prediction models were constructed for both same-species prediction and inter-species prediction.

We investigated the distribution of ORs and the lengths of selected sequence determinants from the LASSO approach (‘LASSO-selected sequence determinants’), and from same-species prediction. The same-species prediction model of human enhancers and promoters had a total of 4321 and 1343 LASSO selected sequence determinants, respectively (S6 and S7 Table). Consistent with the results from the exhaustive search, marginal ORs from the enhancer models were significantly lower than those from the promoter models in all species (Wilcoxon test, *P* < 10^−15^, S2 Fig).

We investigated the relative frequencies of individual LASSO-selected sequence determinants in foreground and background regions, shown as density plots in S3 Fig. In promoters, marginal density of the relative frequencies of LASSO-selected sequence determinants is highly distinct from that of the background, which is consistent with the high effect size of LASSO-selected promoter sequence determinants. On the other hand, marginal densities of LASSO-selected enhancer sequence determinants are similar to those in the background. This observation indicates that in addition to having weaker marginal effects than promoter sequence determinants, the frequency distribution of enhancer sequence determinants is similar between foreground and background.

Interestingly, LASSO selected sequence determinants were significantly longer for enhancers than for promoters (mean lengths of 9.22 in enhancers and 8.32 in promoters in human, *P* <1×10^−15^ by two sample *t*-test, S6 and S7 Table). This pattern was consistent in other species (*P* < 10^−15^ by two-sample *t*-test in all cases). When we applied LASSO approach to 10,000 common sequence determinants, we observed similarly significant differences of effect size and length between enhancer and promoter sequence determinants (S2 and S4 Figs).

### Distinctive effects of GC content and TFBS frequency on enhancer and promoter sequence determinants

**Fig 4:**
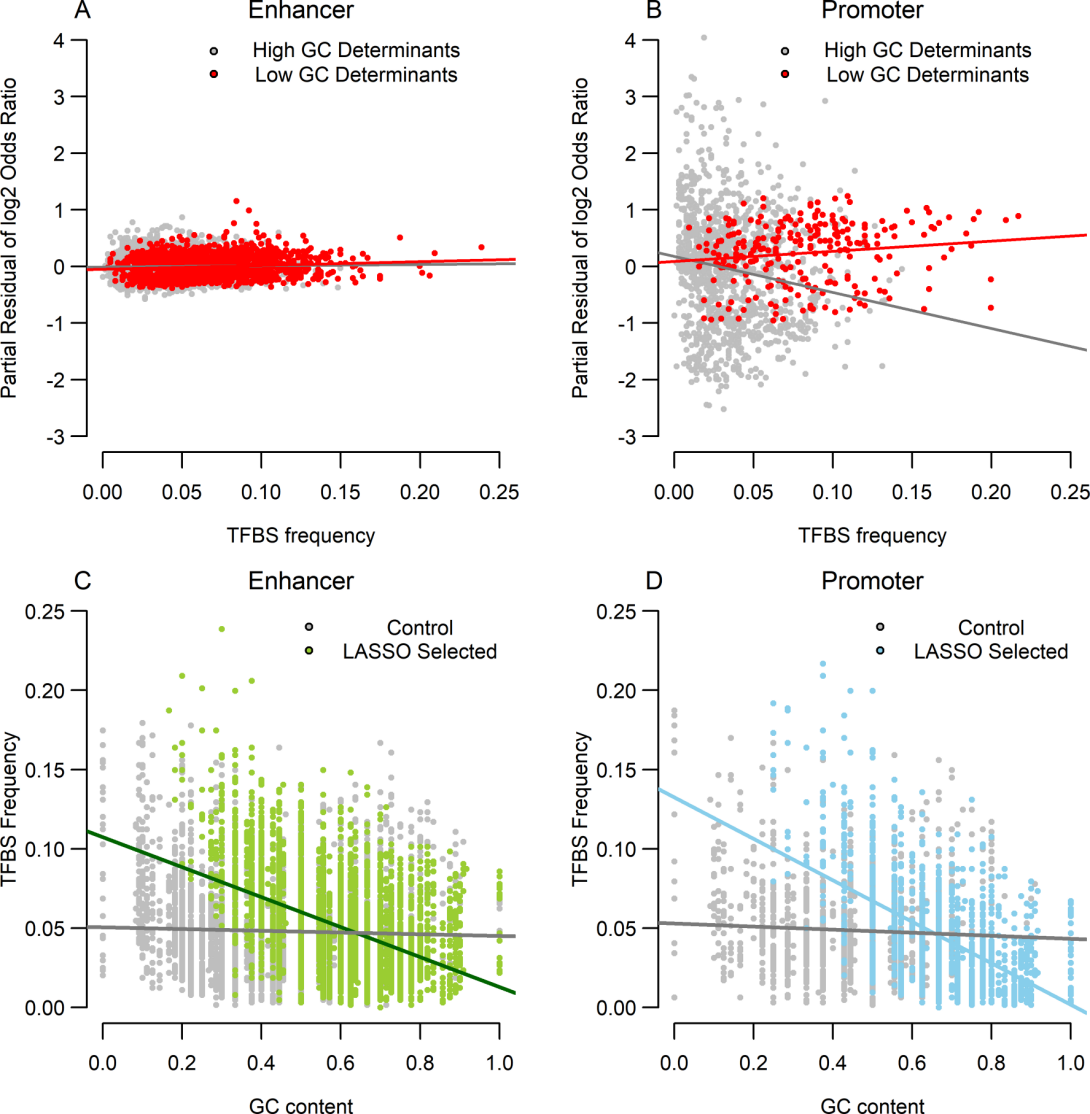
Relationships between several biological variables using LASSO selected sequence determinants from human. First, we present the results from our linear model in Table 3 ((A): Enhancer model results, (B): Promoter model results). To demonstrate the relationship between log_2_OR and TFBS frequency, we regressed out GC content on log_2_OR and drew scatterplots between the resulting partial residual of log_2_OR and TFBS frequency. We further separated the points into two groups, above and below 0.5 GC content. As seen in Fig 4(A) and 4(B), a clear interaction effect was detected only in the promoter model, and TFBS frequency for low GC content is positively correlated with log_2_OR in both models, although the positive correlation is clearer in the promoter model (R^2^: 0.012 and 0.021, P-value: 2.7×10^−5^ and 0.026, for enhancer and promoter, respectively). In Figs 4(C) and 4(D), the negative relationships between GC content and TFBS frequency in enhancers and promoters are depicted in comparison to the background. The green and blue points are results from LASSO selected sequence determinants, while the gray points are control data sets consisting of randomly selected sequence fragments that are not sequence determinants.

We examined two aspects of sequence determinants to understand what features affect enhancer and promoter potentials of specific sequence fragments. Specifically, we used a linear model to analyze the effect of the frequency of G and C nucleotides (GC content) and the frequency of transcription factor binding sites (TFBS frequency). The effect sizes of sequence determinants were response variables, and GC content, TFBS frequency, and their interaction term were explanatory variables. When we analyzed the results of the LASSO-selected sequence determinants, several patterns became clear. First, this model explained a large amount of variation observed in promoter sequence determinants, but only a modest portion of those in enhancer sequence determinants (Table 3). Nevertheless, we found that main factors of GC content and TFBS frequency were positively correlated with the log_2_-transformed OR of sequence determinants both in enhancers and promoters (Table 3, S8 and S9 Tables). However, interaction terms between the two main factors were significantly negative only in promoters. Thus, while GC content and TFBS frequency worked additively to determine the strength of regulatory potential for enhancer sequence determinants, these two factors were antagonistic with each other in promoter sequence determinants (Fig 4(A) and 4(B)). This observation is consistent with previous studies that found a lack of transcription factor binding enrichment at GC-rich promoters compared to GC-poor promoters [51]. We also evaluated–log_10_min(P) instead of TFBS frequency to evaluate the best match between a k-mer and the motifs in the database, and obtained highly similar results for the same models (S10 Table).

**Table 3:** Linear model results of log_2_OR ~ GC content + TFBS frequency + GC content × TFBS frequency + ε.

Dataset	Region	Variable	Estimate	Standard error	P-value	SSR
Human	Enhancer (n = 4321, R^2^ = 0.127)	GC contents	0.56	0.024	< 1×10^−15^	0.11
		TFBS frequency	0.44	0.11	0.00012	0.003
		GC × TFBS	NS			
	Promoter (n = 1342, R^2^ = 0.360)	GC contents	6.40	0.30	< 1×10^−15^	0.21
		TFBS frequency	22.65	2.74	< 1×10^−15^	0.03
		GC × TFBS	-41.84	4.61	< 1×10^−15^	0.04
Mouse	Enhancer (n = 4423, R^2^ = 0.0287)	GC contents	0.25	0.026	< 1×10^−15^	0.020
		TFBS frequency	0.87	0.097	< 1×10^−15^	0.018
		GC × TFBS	NS			
	Promoter (n = 1615, R^2^ = 0.372)	GC contents	5.23	0.23	< 1×10^−15^	0.21
		TFBS frequency	18.79	1.89	< 1×10^−15^	0.038
		GC × TFBS	-37.82	3.09	< 1×10^−15^	0.059

We used LASSO-selected species sequence determinants for these analyses. NS indicates that the interaction terms were not statistically significant at *P* = 0.05. In such cases we conducted log_2_OR ~ GC content + TFBS frequency + ε model instead of the original model. Numbers of sequence determinants, R^2^ values of the models, and Type III partial sum of square in regression (SSR) for each variable are also provided.

In summary, TFBS frequency was positively correlated with effect size in both of enhancer and promoters when GC content was low. On the other hand, the estimated coefficients of GC content and TFBS frequency were higher in promoters than in enhancers, indicating that the effects of these factors were stronger in promoters compared to in enhancers. Accordingly, the R^2^ of the linear models were substantially higher for promoters than for enhancers (Table 3, S8 and S9 Table). Second, the relationships between GC contents and TFBS frequency were negative in both of enhancer and promoter analysis (Fig 4(C) and 4(D)). Accordingly, sequence determinants that were GC-rich tended to lack TFBS, and low GC sequence determinants tended to harbor more TFBS than high GC sequences [51]. The whole set of sequence determinants obtained from exhaustive search yielded similar results (S11 Table).

### LASSO prediction models can be inter-changed between species

**Fig 5:**
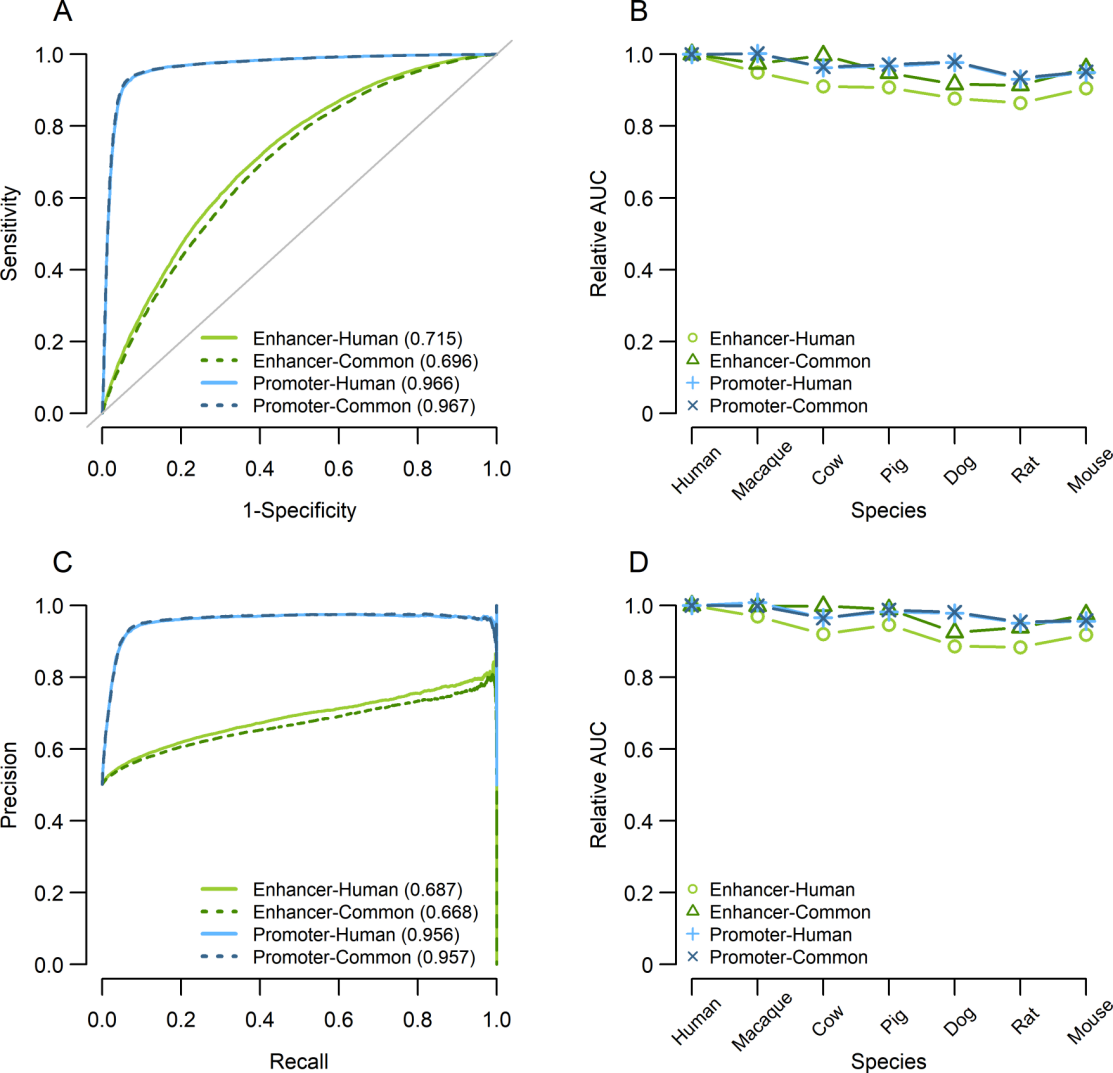
Prediction accuracies of LASSO models as measured by AUCs. (A) ROC curves of the human same-prediction result from ten-fold cross validation. Solid lines represent prediction with human species sequence determinants, while dashed lines represent those with common sequence determinants. (B) Cross-species prediction AUCs based on human prediction models. The Y-axis represents relative AUC value calculated as the ratio between cross-species prediction AUC and same-species prediction AUC based on the human models constructed using human species sequence determinants (circle or cross marks) and common sequence determinants (triangle or “x” marks), respectively. (C) PR curves of the human same-prediction result from ten-fold cross validation. (D) Cross-species prediction results PR-AUCs based on human prediction models.

The prediction accuracy of the human promoter same-species prediction model was very high, with an AUC of 0.97 (Fig 5). Same-species prediction models from other six species exhibited similarly high AUCs (S12 and S13 Table), indicating that promoters can be accurately predicted from sequence determinants. We also evaluated prediction AUCs using 10,000 non-sequence determinants, while matching the distributions of GC content and length as those of sequence determinants. We then constructed prediction models using LASSO for enhancers and promoters in human and mouse, respectively. We iterated the process five times to measure variability of the AUCs. Results are shown in S6 Fig. The AUCs of models using non-sequence determinants were lower than AUCs with sequence determinants. For example, human and mouse enhancer prediction AUCs with non-sequence determinants showed 0.507 and 0.002, and 0.500 and 0.007 for mean and standard deviation, respectively. These results indicate that non-sequence determinants had poor prediction performances. In case of promoters, the mean and standard deviation of AUCs were 0.636 and 0.006 for human, and 0.608 and 0.004 for mouse, respectively. These values were higher than those of enhancers, likely reflecting the effect of GC contents (e.g., [52]). Nevertheless, they were substantially lower than the AUCs with sequence determinants, indicating that sequence determinants have superior prediction performances than non-sequence determinants.

Next, we tested if prediction models constructed from one species could be used in different species, to investigate if different genomes use similar sequence determinants to encode promoters. Indeed, when we calculated AUCs of inter-species prediction between seven species of promoters, the AUCs were all above 0.9, indicating high accuracy (Fig 5).

On the other hand, the LASSO prediction models of enhancers had the following differences from those of promoters. First, the enhancer models using 10,000 species determinants had 2.5- to 4.2-fold greater numbers of explanatory variables than the promoter models (S12 Table). However, their AUCs were generally lower than those of the promoter models (Fig 5). We found that same-species prediction AUCs for enhancer models were greater than 0.7, and the highest was when mouse model were used to predict mouse enhancers, 0.76 (S12 Table). Nevertheless, inter-species prediction results using enhancer models showed similar AUCs to same-species enhancer predictions (Fig 5).

We tested if the high inter-species prediction accuracies were driven by the presence of highly conserved regulatory elements across different mammalian species. The proportions of conserved enhancer regions among the seven species were much smaller than those of promoter regions, as previously described [6] (Table 1). Interestingly, we observed similar AUCs before and after removing highly conserved regulatory regions at both enhancers and promoters (S14 Table), suggesting that conserved regulatory regions were not responsible for the high predictabilities across species. We then extracted 10 subsets of 10,000 sequence determinants from human enhancer and promoter sequence determinants (all subsets were mutually exclusive with each other subset) and constructed LASSO models to apply to the same-species (human) prediction and inter-species (mouse enhancer) prediction. We found that the AUCs of these 10 subsets were highly similar (S6 Fig). Thus, even though the regulatory regions themselves were not conserved in terms of their precise location, mammalian enhancers and promoters have inter-changeability in terms of prediction between species.

We also constructed LASSO models using 10,000 common sequence determinants from all seven species. AUC values for promoter prediction were highly similar to those obtained from models using species sequence determinants (Fig 5, S13 Table), indicating that sequence fragments that were commonly enriched in all 7 species harbor sufficient signals for promoter prediction. On the other hand, enhancer prediction results using 10,000 common sequence determinants showed slight decrease of AUC compared to same-species prediction (mean AUC of prediction with species determinants: 0.723, that with common determinants: 0.679). Interestingly, mean numbers of LASSO selected common sequence determinants were significantly lower than the species ones in enhancers (1613 and 3970 for common sequence determinants and species sequence determinants, respectively; *P* <1×10^−5^ by paired *t*-test), while they were not significantly different in promoter models (1138 and 1342 for common sequence determinants and species sequence determinants, respectively; *P* = 0.2114 by paired *t*-test). This implies that each of the common enhancer sequence determinants may have higher predictive capabilities than species sequence determinants.

### Impact of sequence composition and prediction performance

While background and foreground of enhancers exhibit similar GC distribution, foreground regions of promoters are substantially skewed towards GC-rich regions (the average difference was 10.0%, higher in promoters than in enhancers) (S7 Fig). Therefore, we investigated how GC content difference between foreground and background might affect prediction analyses. First, to measure the impact of GC content alone in prediction performances, we calculated AUCs using only GC content as a predictor (Table 4). Second, we constructed LASSO models using sequence determinants of low-GC content (GC content ≤0.5) to measure prediction performances without effects of high GC content sequence determinants. For this analysis, we randomly selected 10,000 sequence determinants with stratification of GC content and sequence length. These results were then compared to those of the original AUCs.

**Table 4:** Comparisons between the original AUC to those obtained using low-GC sequence determinants (AUC with low-GC sequence determinants), and GC content only (AUC with GC content).

	Enhancer			Promoter		
	Original AUC	AUC with low-GC sequence determinants	AUC with GC content	Original AUC	AUC with low-GC sequence determinants	AUC with GC content
Human	0.715	0.732	0.579	0.966	0.941	0.861
Macaque	0.713	0.723	0.600	0.967	0.933	0.873
Cow	0.714	0.712	0.640	0.938	0.897	0.803
Pig	0.702	0.724	0.577	0.946	0.889	0.830
Dog	0.717	0.724	0.571	0.949	0.896	0.843
Rat	0.741	0.736	0.560	0.916	0.856	0.818
Mouse	0.756	0.768	0.593	0.929	0.886	0.830
Average	0.723	0.731	0.589	0.944	0.900	0.837

We found the AUCs using only GC content reflected the amount of GC content differences between foreground and background (S7 Fig). For example, average AUCs using only GC content were 0.589 and 0.837 in enhancers and promoters, respectively. However, both of those AUCs were considerably lower than the original AUCs (differences of 0.134 and 0.107 in enhancers and promoters, respectively), meaning that GC content could not explain all of the variation between foreground and background. This observation is consistent with a prior study utilizing a similar approach [52]. Moreover, models with low-GC sequence determinants had higher AUCs than those using only GC contents. In other words, models without high GC content sequence determinants outperformed the AUCs with only GC contents. Interestingly, mean AUCs with low-GC sequence determinants in enhancers were even higher than those of the original AUCs, which may imply that low-GC enhancers sequence determinants had better prediction performances than high-GC sequence determinants when they were jointly used for prediction. In conclusion, prediction performances of the sequence determinants detected by LASSO cannot be attributed to their GC contents.

## Discussion

Understanding specific histone modifications marking enhancers and promoters has opened the way to identify these regions using ChIP-seq, which complements and scales up traditional transcription factor binding assays [1, 6, 53]. Even though our understanding of the exact molecular nature of regulatory regions continues to improve, technical advances in epigenomic assays have opened a new opportunity to study evolution of regulatory regions using unbiased genome-wide epigenomic profiling. We were motivated by two observations: that regulatory regions identified from epigenomic assays can be predicted with high accuracy in case of same-species prediction [7–11], yet that they are highly divergent between different species [6, 18, 20, 21]. The fact that regulatory regions can be predicted with high accuracy implies that specific sequence fragments can encode regulatory function. Indeed, previous studies often referred to such fragments as *cis*-regulatory motifs. Since they encode function, they are likely to be subject to natural selection (largely purifying selection) and thus evolutionarily conserved. However, genome-wide studies indicate that regulatory regions, especially enhancers, are highly divergent between species. To investigate this potentially paradoxical pattern of evolution of regulatory regions, we used a powerful approach to examine every possible sequence fragments for their statistical enrichment in experimentally verified enhancers and promoters of seven mammalian species. This approach, which we named exhaustive search, revealed that numerous sequence fragments were statistically over-represented in enhancers and promoters (which we named as sequence determinants).

Sequence determinants underlying enhancers and promoters exhibited intriguing differences with respect to their degree of enrichment (effect size), GC content, and the frequencies of known TFBS. Notably, the degree of statistical enrichment was significantly higher for promoter sequence determinants compared to enhancer sequence determinants. This observation suggests that sequence determinants may have greater impacts on the regulatory potential of promoters than of enhancers. This idea is also consistent with the fact that promoters are more evolutionarily conserved than enhancers [6].

We next applied a machine-learning method, LASSO, to reduce interdependence among sequence determinants and construct prediction models based on the non-redundant sequence determinant set. Same-species prediction models generated from these sequence determinants had high AUCs for enhancers and promoters (Fig 5 and S12 and S13 Tables), affirming the predictor power of sequence determinants [11, 52]. The AUCs from these models are on par with those from previous studies that utilized different approaches (e.g., [11]). We observed that enhancer models utilized greater numbers of predictors yet exhibited lower accuracy compared to promoter models, which can be explained by promoter sequence determinants associated with significantly higher effect sizes compared to enhancer sequence determinants (Figs 2 and 3, S2 and S4 Figs). Furthermore, we applied prediction models generated from one mammal to other mammals, to directly test whether sequence determinants from one species could be used to predict regulatory regions in other species. Remarkably, even though the sequence determinants themselves had only moderate overlaps between species (S5 Table), models constructed from one species could predict promoters in other species with high accuracies (S12 and S13 Tables). As for enhancer models, AUCs from inter-species prediction models were also comparable to same-species predictions (Fig 5). In other words, the extent to which prediction models could be inter-changed between species was similar between enhancers and promoters (Fig 5).

We used a cutoff effect size for sequence determinants as 1, for the following reasons. First, many sequence determinants have extremely low p-values despite low effect sizes due to their abundance, especially those with shorter lengths. For example, 25% of human enhancer sequence determinants among those of top 10,000 lowest p-values have effect sizes smaller than 1.2. Second, when we constructed a human enhancer prediction model using randomly selected 10,000 sequence determinants with effect sizes smaller than 1.2, the resulting AUC was 0.715, which is equivalent to the original AUC. Moreover, when we applied this model to mouse, the inter-species AUC was 0.680, even higher than the original AUC (0.647). Therefore, setting an arbitrary cutoff value is likely to result in the loss of true sequence determinants that are important in terms of prediction performances.

Integrating the main findings that 1) there are a large number of sequence determinants that potentially contribute to the regulatory roles of enhancers and promoters; 2) the strength of statistical enrichment of sequence determinant is greater for promoters, which are more evolutionarily conserved than enhancers; 3) prediction accuracies of models generated using sequence determinants from different species are comparable to each other, we hypothesize the following. Even though the specific motifs that encode regulatory regions are different between species [6, 18, 20, 21], the function of specific sequence determinants could be conserved between species. There may exist a large reservoir of potential sequence determinants that can contribute to regulatory regions of many species.

## Supporting information

S1 Fig Changes of AUC according to the number of input sequence determinants. (TIF)

S2 Fig Distribution of effect sizes (ORs) from the LASSO. We drew boxplots of effect sizes of the LASSO selected determinants for species determinants (A), and common determinants (B). In general, effect sizes from the enhancer analysis are smaller than those from the promoter analysis.
(TIF)

S3 Fig Distribution of relative frequencies of sequence determinants in foreground and background regions. The X-axis is log_10_ transformed relative frequency and Y-axis is density of the relative frequency. FG and BG in the figure legends stand for foreground and background, respectively.
(TIF)

S4 Fig Heatmaps of the LASSO selected sequence determinant counts for human, according to their marginal OR and sequence length. X-axis of the heatmaps is the length of sequence determinants and Y-axis of those is OR of each sequence determinants. (A) and (B) are from human species sequence determinants and (C) and (D) are from common sequence determinants. Note that the counts of species sequence determinants in the other six species are summarized in S6 and S7 Table.
(TIF)

S5 Fig AUCs from LASSO prediction models using non-sequence determinants with matching GC content and sequence length as true sequence determinants. We applied LASSO and iterated the process five times. Colored points are AUCs using same number of matched sequence determinants. AUCs with sequence determinants are clearly higher than those with non-sequence determinants.
(TIF)

S6 Fig AUCs using 10 subsets of sequence determinants of human enhancer (green) and promoter (blue) for same species prediction (filled lines) and inter species prediction (dashed lines). Mean and standard deviation of each set of the 10 replicated AUCs are also shown.
(TIF)

S7 Fig Distribution of GC content of the regulatory regions (enhancers in green and promoters in blue) and their correspondent control regions (dashed lines). (TIF)

S1 Table Overlapped proportions of foreground and background regions. (PDF)

S2 Table Summary of enhancer species sequence determinants in seven species. Enhancer sequence determinants are generally concentrated in the low OR ranges.
(PDF)

S3 Table Summary of promoter species sequence determinants in seven species. Promoter sequence determinants are generally concentrated in the high OR ranges.
(PDF)

S4 Table Summary of common sequence determinants determined from the CMH test. Numbers of intersecting significant species sequence determinants from all seven species are shown in parentheses. Note that the ORs in this table represent the minimum ORs among the seven species.
(PDF)

S5 Table The proportion of species sequence determinants that are also found in common sequence determinants. There are few enhancer sequence determinants with large effect size that are common in the 7 mammalian species. On the other hand, the proportion of overlapping promoter sequence determinants is consistent across different effect sizes.
(PDF)

S6 Table Counts of LASSO-selected enhancer sequence determinants. The numbers outside the parentheses are from LASSO with species sequence determinants, while those in the parentheses are from LASSO with common sequence determinants.
(PDF)

S7 Table Counts of LASSO-selected promoter sequence determinants. The numbers outside the parentheses are from LASSO with species sequence determinants, while those within the parentheses are from LASSO with common sequence determinants.
(PDF)

S8 Table Results of linear model analyses from five mammalian species. The model used is: log_2_OR ~ GC contents + TFBS Frequency + GC contents × TFBS Frequency + ε. NS indicates that the interaction terms were not statistically significant at *P* = 0.05. In such cases, we conducted log_2_OR ~ GC contents + TFBS Frequency + GC contents + ε model instead. R^2^ values of the models are also provided.
(PDF)

S9 Table Linear model results of log_2_OR ~ GC contents + TFBS Frequency + GC contents × TFBS Frequency + ε. The data sets used in this analysis are from results of LASSO selected common sequence determinants. Test results whose interaction terms are NS indicate that the interaction terms were not statistically significant at *P* = 0.05, and we conducted log_2_OR ~ GC contents + TFBS Frequency + GC contents + ε model instead. R^2^ values of the models are also provided.
(PDF)

S10 Table Linear model results of log_2_OR ~ GC content + -log_10_min(P) + GC content × -log_10_min(P) + ε. We used same models that were used in Table 3 and substituted -log_10_min(P) for TFBS frequency.
(PDF)

S11 Table Linear model results of log_2_OR ~ GC contents + TFBS Frequency + GC contents × TFBS Frequency + ε. The data sets used in this analysis are from results of exhaustive search. When interaction term of the model was not significant (NS), we conducted log_2_OR ~ GC contents + TFBS Frequency + ε model instead.
(PDF)

S12 Table Prediction results using LASSO approach and 10,000 species sequence determinants for enhancers and promoters. The 10,000 determinants were selected using stratified random sampling from the exhaustive search results. Columns refer to LASSO models trained for the seven species, and rows show test data sets to be predicted by the LASSO trained models. The values in parenthesis under the species names indicate the number of LASSO selected sequence determinants of enhancers (left) and promoters (right). AUC values out of parenthesis are receiver operating characteristic (ROC)-AUCs and those in parenthesis are precision-recall (PR)-AUCs. Note that the AUC values in diagonal terms are same-species prediction AUC and the other values in off-diagonal terms are inter-species prediction AUC values.
(PDF)

S13 Table Prediction result using the LASSO approach and 10,000 common sequence determinants in enhancers and promoters. The 10,000 determinants were selected using stratified random sampling from the exhaustive search results. Columns of the table are LASSO models that were trained from the seven species, and rows are test data sets to be predicted by the LASSO trained models. The values in parentheses under the species names are the number of LASSO selected sequence determinants of enhancers (left) and promoters (right). AUC values outside of parenthesis are receiver operating characteristic (ROC)-AUCs and those in parenthesis are precision-recall (PR)-AUCs. Note that the AUC values in diagonal terms are same-species prediction AUC and the other values in off-diagonal terms are inter-species prediction AUC values.
(PDF)

S14 Table Prediction results using human and mouse data sets without globally conserved regions. Using the information described in Table 1, we conducted the same procedure of exhaustive search and LASSO prediction using 10K species sequence determinants. We then calculated AUCs and compared them to the original analysis results.
(PDF)

## References

[1] Allis CD, Jenuwein T. The molecular hallmarks of epigenetic control. Nature Reviews Genetics. 2016. 10.1038/nrg.2016.59 27346641

[2] Creyghton MP, Cheng AW, Welstead GG, Kooistra T, Carey BW, Steine EJ, et al Histone H3K27ac separates active from poised enhancers and predicts developmental state. Proceedings of the National Academy of Sciences. 2010;107(50):21931–6. 10.1073/pnas.1016071107 PMC3003124 21106759

[3] Nord AS, Blow MJ, Attanasio C, Akiyama JA, Holt A, Hosseini R, et al Rapid and pervasive changes in genome-wide enhancer usage during mammalian development. Cell. 2013;155(7):1521–31. 10.1016/j.cell.2013.11.033 24360275 PMC3989111

[4] Cain CE, Blekhman R, Marioni JC, Gilad Y. Gene expression differences among primates are associated with changes in a histone epigenetic modification. Genetics. 2011;187(4):1225–34. 10.1534/genetics.110.126177 21321133 PMC3070530

[5] Santos-Rosa H, Schneider R, Bannister AJ, Sherriff J, Bernstein BE, Emre NT, et al Active genes are tri-methylated at K4 of histone H3. Nature. 2002;419(6905):407–11. 10.1038/nature01080 12353038

[6] Villar D, Berthelot C, Aldridge S, Rayner TF, Lukk M, Pignatelli M, et al Enhancer evolution across 20 mammalian species. Cell. 2015;160(3):554–66. 10.1016/j.cell.2015.01.006 ; PubMed Central PMCID: PMCPMC4313353. 25635462 PMC4313353

[7] Rouault H, Mazouni K, Couturier L, Hakim V, Schweisguth F. Genome-wide identification of cis-regulatory motifs and modules underlying gene coregulation using statistics and phylogeny. Proc Natl Acad Sci U S A. 2010;107(33):14615–20. 10.1073/pnas.1002876107 ; PubMed Central PMCID: PMCPMC2930411. 20671200 PMC2930411

[8] Zou C, Sun K, Mackaluso JD, Seddon AE, Jin R, Thomashow MF, et al Cis-regulatory code of stress-responsive transcription in Arabidopsis thaliana. Proceedings of the National Academy of Sciences. 2011;108(36):14992–7. 10.1073/pnas.1103202108 PMC3169165 21849619

[9] Dubos C, Kelemen Z, Sebastian A, Bülow L, Huep G, Xu W, et al Integrating bioinformatic resources to predict transcription factors interacting with cis-sequences conserved in co-regulated genes. BMC genomics. 2014;15(1):317. 24773781 10.1186/1471-2164-15-317 PMC4234446

[10] Yuan G-C, Liu JS. Genomic sequence is highly predictive of local nucleosome depletion. PLoS computational biology. 2008;4(1):e13 10.1371/journal.pcbi.0040013 18225943 PMC2211532

[11] Whitaker JW, Chen Z, Wang W. Predicting the human epigenome from DNA motifs. Nature methods. 2015;12(3):265–72. 10.1038/nmeth.3065 25240437 PMC4344378

[12] Dermitzakis ET, Clark AG. Evolution of transcription factor binding sites in Mammalian gene regulatory regions: conservation and turnover. Molecular biology and evolution. 2002;19(7):1114–21. 10.1093/oxfordjournals.molbev.a004169 12082130

[13] Odom DT, Dowell RD, Jacobsen ES, Gordon W, Danford TW, MacIsaac KD, et al Tissue-specific transcriptional regulation has diverged significantly between human and mouse. Nature genetics. 2007;39(6):730–2. 10.1038/ng2047 17529977 PMC3797512

[14] Schmidt D, Wilson MD, Ballester B, Schwalie PC, Brown GD, Marshall A, et al Five-vertebrate ChIP-seq reveals the evolutionary dynamics of transcription factor binding. Science. 2010;328(5981):1036–40. 10.1126/science.1186176 20378774 PMC3008766

[15] Boffelli D, McAuliffe J, Ovcharenko D, Lewis KD, Ovcharenko I, Pachter L, et al Phylogenetic shadowing of primate sequences to find functional regions of the human genome. Science. 2003;299(5611):1391–4. 10.1126/science.1081331 12610304

[16] Margulies EH, Vinson JP, Miller W, Jaffe DB, Lindblad-Toh K, Chang JL, et al An initial strategy for the systematic identification of functional elements in the human genome by low-redundancy comparative sequencing. Proceedings of the National Academy of Sciences of the United States of America. 2005;102(13):4795–800. 10.1073/pnas.0409882102 15778292 PMC555705

[17] Prabhakar S, Poulin F, Shoukry M, Afzal V, Rubin EM, Couronne O, et al Close sequence comparisons are sufficient to identify human cis-regulatory elements. Genome research. 2006;16(7):855–63. 10.1101/gr.4717506 16769978 PMC1484452

[18] Pennacchio LA, Ahituv N, Moses AM, Prabhakar S, Nobrega MA, Shoukry M, et al In vivo enhancer analysis of human conserved non-coding sequences. Nature. 2006;444(7118):499–502. 10.1038/nature05295 17086198

[19] Prescott SL, Srinivasan R, Marchetto MC, Grishina I, Narvaiza I, Selleri L, et al Enhancer divergence and cis-regulatory evolution in the human and chimp neural crest. Cell. 2015;163(1):68–83. 10.1016/j.cell.2015.08.036 26365491 PMC4848043

[20] Shibata Y, Sheffield NC, Fedrigo O, Babbitt CC, Wortham M, Tewari AK, et al Extensive evolutionary changes in regulatory element activity during human origins are associated with altered gene expression and positive selection. PLoS genetics. 2012;8(6):e1002789 10.1371/journal.pgen.1002789 22761590 PMC3386175

[21] Degner JF, Pai AA, Pique-Regi R, Veyrieras J-B, Gaffney DJ, Pickrell JK, et al DNase [thinsp] I sensitivity QTLs are a major determinant of human expression variation. Nature. 2012;482(7385):390–4. 10.1038/nature10808 22307276 PMC3501342

[22] Pierstorff N, Bergman CM, Wiehe T. Identifying cis-regulatory modules by combining comparative and compositional analysis of DNA. Bioinformatics. 2006;22(23):2858–64. 10.1093/bioinformatics/btl499 17032682

[23] Bush EC, Lahn BT. A genome-wide screen for noncoding elements important in primate evolution. BMC Evolutionary Biology. 2008;8(1):17 10.1186/1471-2148-8-17 18215302 PMC2242780

[24] Moses AM, Pollard DA, Nix DA, Iyer VN, Li X-Y, Biggin MD, et al Large-scale turnover of functional transcription factor binding sites in Drosophila. PLOS Computational Biology. 2006;2(10):e130 10.1371/journal.pcbi.0020130 17040121 PMC1599766

[25] Ludwig MZ, Bergman C, Patel NH, Kreitman M. Evidence for stabilizing selection in a eukaryotic enhancer element. Nature. 2000;403:564 10.1038/35000615 10676967

[26] Tsong AE, Tuch BB, Li H, Johnson AD. Evolution of alternative transcriptional circuits with identical logic. Nature. 2006;443(7110):415 10.1038/nature05099 17006507

[27] Ihmels J, Bergmann S, Gerami-Nejad M, Yanai I, McClellan M, Berman J, et al Rewiring of the yeast transcriptional network through the evolution of motif usage. Science. 2005;309(5736):938–40. 10.1126/science.1113833 16081737

[28] Lynch VJ, Leclerc RD, May G, Wagner GP. Transposon-mediated rewiring of gene regulatory networks contributed to the evolution of pregnancy in mammals. Nature genetics. 2011;43(11):1154 10.1038/ng.917 21946353

[29] Venkataram S, Fay JC. Is transcription factor binding site turnover a sufficient explanation for cis-regulatory sequence divergence? Genome biology and evolution. 2010;2:851–8. 10.1093/gbe/evq066 21068212 PMC2997565

[30] Villar D, Flicek P, Odom DT. Evolution of transcription factor binding in metazoans—mechanisms and functional implications. Nature Reviews Genetics. 2014;15(4):221 10.1038/nrg3481 24590227 PMC4175440

[31] Venter JC, Adams MD, Myers EW, Li PW, Mural RJ, Sutton GG, et al The sequence of the human genome. science. 2001;291(5507):1304–51. 10.1126/science.1058040 11181995

[32] Elango N, Kim S-H, Program NCS, Vigoda E, Yi SV. Mutations of different molecular origins exhibit contrasting patterns of regional substitution rate variation. PLoS Computational Biology. 2008;4(2):e1000015 10.1371/journal.pcbi.1000015 18463707 PMC2265638

[33] Friedman J, Hastie T, Tibshirani R. Regularization paths for generalized linear models via coordinate descent. Journal of statistical software. 2010;33(1):1 20808728 PMC2929880

[34] Hebiri M, Lederer J. How correlations influence lasso prediction. IEEE Transactions on Information Theory. 2013;59(3):1846–54.

[35] Usai MG, Goddard ME, Hayes BJ. LASSO with cross-validation for genomic selection. Genetics research. 2009;91(6):427–36. 10.1017/S0016672309990334 20122298

[36] Nekrutenko A, Li W-H. Assessment of compositional heterogeneity within and between eukaryotic genomes. Genome Research. 2000;10(12):1986–95. 11116093 10.1101/gr.10.12.1986 PMC313050

[37] Pages H, Pages MH, SequenceMatching A, GenomeInfoDb G, Biostrings R, SNPlocs-class R. Package ‘BSgenome’. 2015.

[38] Yates A, Beal K, Keenan S, McLaren W, Pignatelli M, Ritchie GR, et al The Ensembl REST API: ensembl data for any language. Bioinformatics. 2014;31(1):143–5. 10.1093/bioinformatics/btu613 25236461 PMC4271150

[39] Spudich GM, Fernández-Suárez XM. Touring Ensembl: a practical guide to genome browsing. BMC genomics. 2010;11(1):295. 20459808 10.1186/1471-2164-11-295 PMC2894802

[40] Benjamini Y, Hochberg Y. Controlling the false discovery rate: a practical and powerful approach to multiple testing. Journal of the royal statistical society Series B (Methodological). 1995:289–300.

[41] Mantel N. Chi-square tests with one degree of freedom; extensions of the Mantel-Haenszel procedure. Journal of the American Statistical Association. 1963;58(303):690–700.

[42] Day N, Byar D. Testing hypotheses in case-control studies-equivalence of Mantel-Haenszel statistics and logit score tests. Biometrics. 1979:623–30. 497345

[43] Scheaffer RL, Mendenhall III W, Ott RL, Gerow KG. Elementary survey sampling: Cengage Learning; 2011.

[44] Stone M. Cross-validatory choice and assessment of statistical predictions. Journal of the royal statistical society Series B (Methodological). 1974:111–47.

[45] McLachlan G, Do K-A, Ambroise C. Analyzing microarray gene expression data: John Wiley & Sons; 2005.

[46] Davis J, Goadrich M, editors. The relationship between Precision-Recall and ROC curves. Proceedings of the 23rd international conference on Machine learning; 2006: ACM.

[47] Zou H, Hastie T. Regularization and variable selection via the elastic net. Journal of the Royal Statistical Society: Series B (Statistical Methodology). 2005;67(2):301–20.

[48] Gupta S, Stamatoyannopoulos JA, Bailey TL, Noble WS. Quantifying similarity between motifs. Genome biology. 2007;8(2):R24 10.1186/gb-2007-8-2-r24 17324271 PMC1852410

[49] Mathelier A, Zhao X, Zhang AW, Parcy F, Worsley-Hunt R, Arenillas DJ, et al JASPAR 2014: an extensively expanded and updated open-access database of transcription factor binding profiles. Nucleic acids research. 2013;42(D1):D142–D7. 24194598 10.1093/nar/gkt997 PMC3965086

[50] Kulakovskiy IV, Vorontsov IE, Yevshin IS, Soboleva AV, Kasianov AS, Ashoor H, et al HOCOMOCO: expansion and enhancement of the collection of transcription factor binding sites models. Nucleic acids research. 2016;44(D1):D116–D25. 10.1093/nar/gkv1249 26586801 PMC4702883

[51] Roider HG, Lenhard B, Kanhere A, Haas SA, Vingron M. CpG-depleted promoters harbor tissue-specific transcription factor binding signals—implications for motif overrepresentation analyses. Nucleic Acids Res. 2009;37(19):6305–15. 10.1093/nar/gkp682 ; PubMed Central PMCID: PMCPMC2770660. 19736212 PMC2770660

[52] Landolin JM, Johnson DS, Trinklein ND, Aldred SF, Medina C, Shulha H, et al Sequence features that drive human promoter function and tissue specificity. Genome research. 2010;20(7):890–8. 10.1101/gr.100370.109 20501695 PMC2892090

[53] Sarda S, Hannenhalli S. Next-Generation Sequencing and Epigenomics Research: A Hammer in Search of Nails. Genomics Inform. 2014;12(1):2–11. 10.5808/GI.2014.12.1.2 24748856 PMC3990762

