# Supplementary figures and images for: Functional conservation of sequence determinants at rapidly evolving regulatory regions across mammals
Source: PLoS Comput Biol. 2018 Oct 5;14(10):e1006451. doi: 10.1371/journal.pcbi.1006451 (PMC6192654; doi:10.1371/journal.pcbi.1006451)

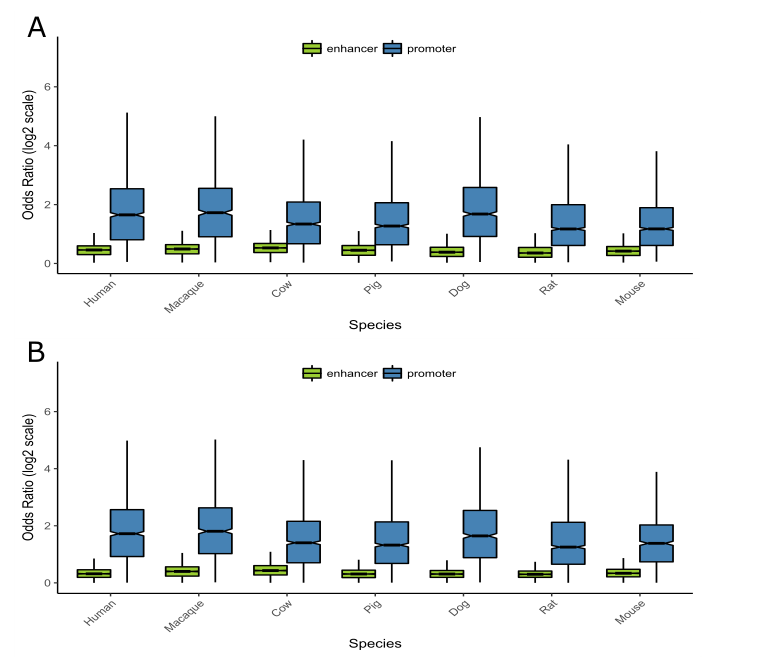

Supplement: S2 Fig — We drew boxplots of effect sizes of the LASSO selected determinants for species determinants (A), and common determinants (B). In general, effect sizes from the enhancer analysis are smaller than those from the promoter analysis. (TIF) [file pcbi.1006451.s002.tif]

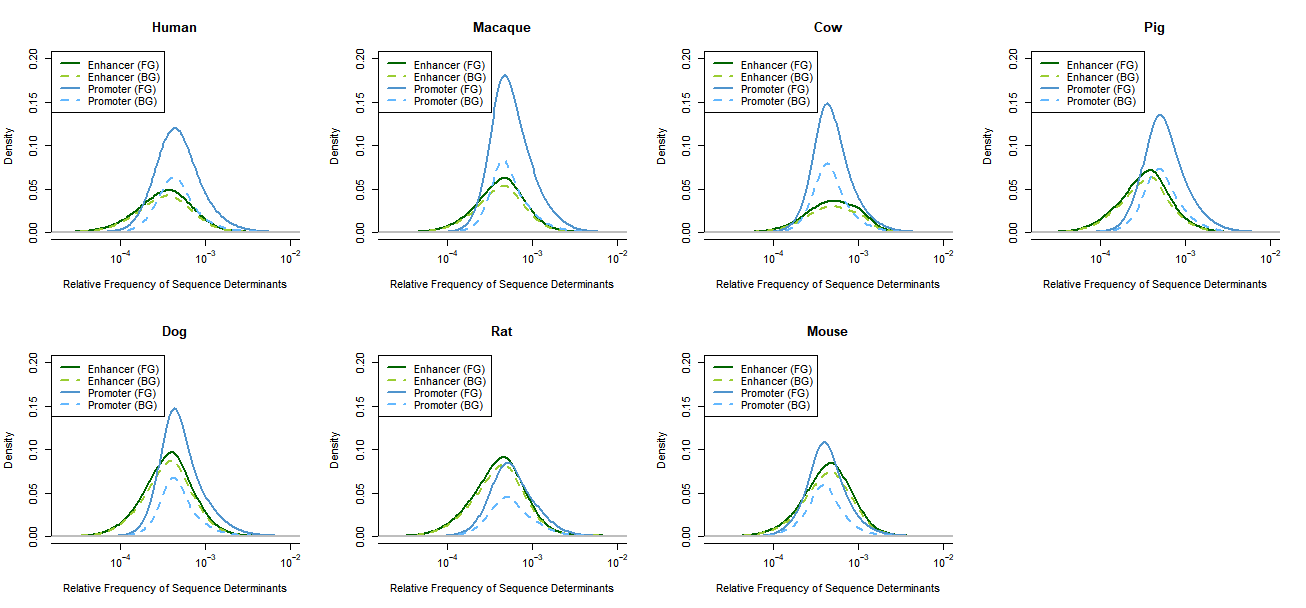

Supplement: S3 Fig — The X-axis is log10 transformed relative frequency and Y-axis is density of the relative frequency. FG and BG in the figure legends stand for foreground and background, respectively. (TIF) [file pcbi.1006451.s003.tif]

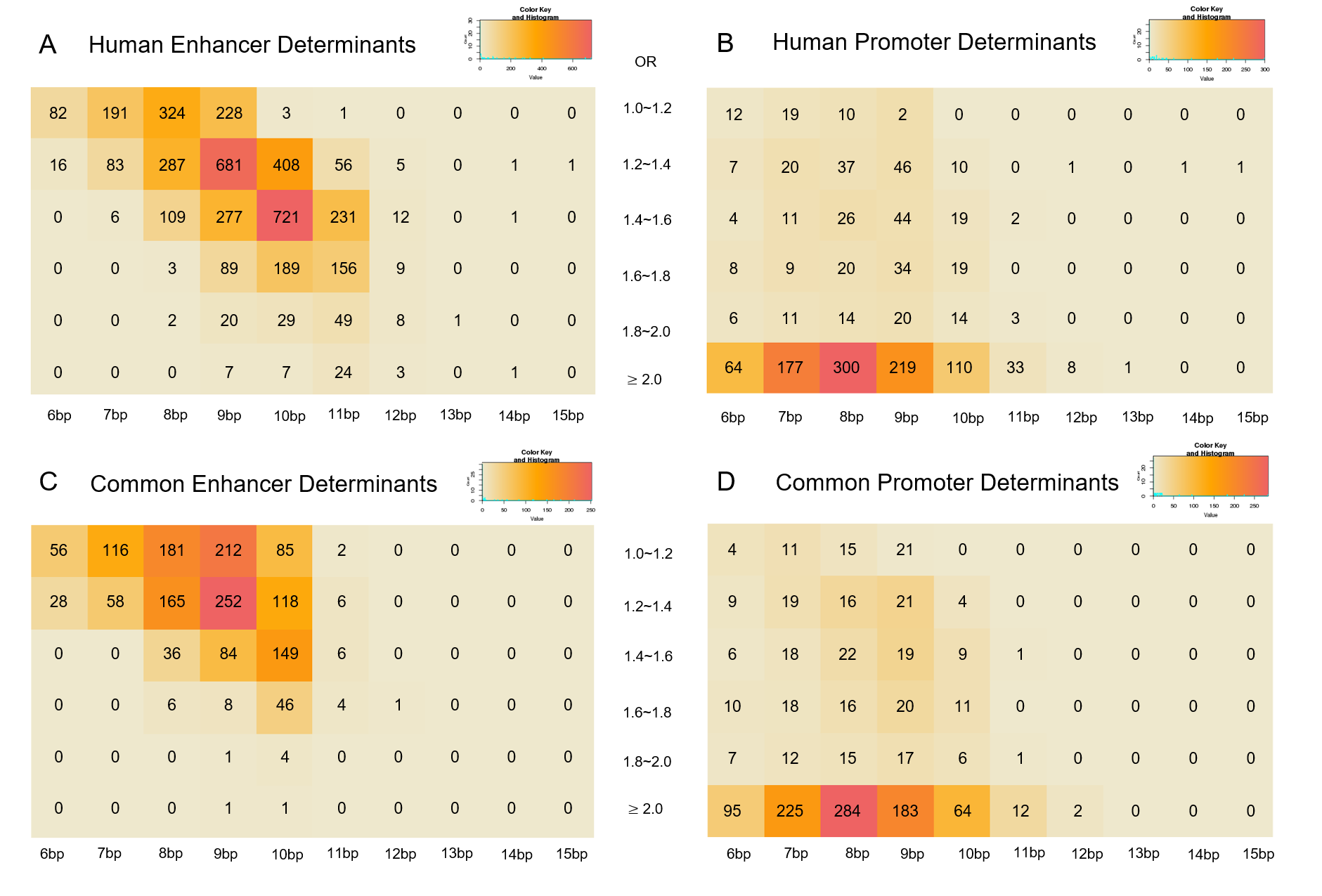

Supplement: S4 Fig — X-axis of the heatmaps is the length of sequence determinants and Y-axis of those is OR of each sequence determinants. (A) and (B) are from human species sequence determinants and (C) and (D) are from common sequence determinants. Note that the counts of species sequence determinants in the other six species are summarized in S6 and S7 Table. (TIF) [file pcbi.1006451.s004.tif]

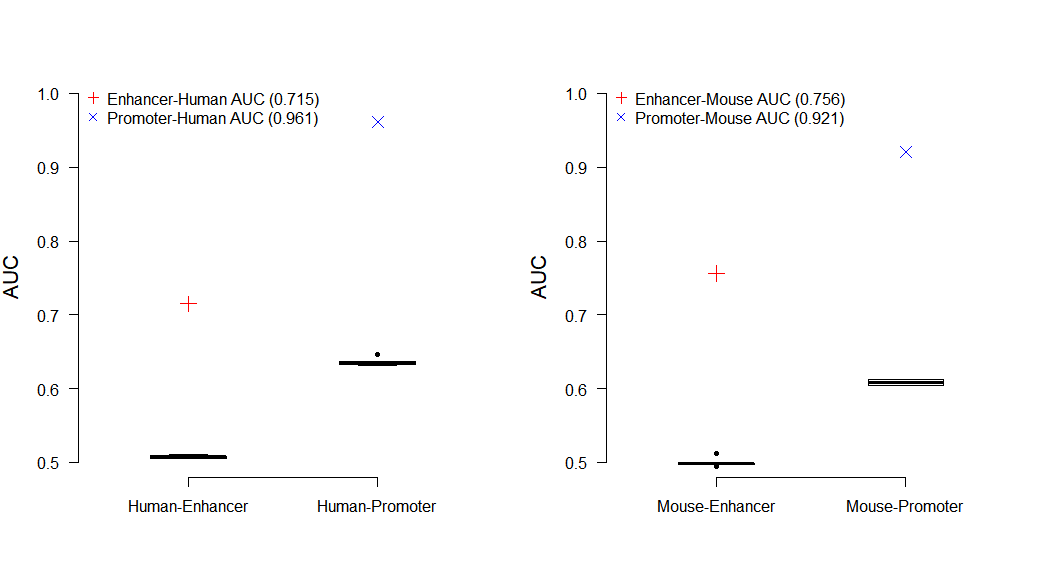

Supplement: S5 Fig — We applied LASSO and iterated the process five times. Colored points are AUCs using same number of matched sequence determinants. AUCs with sequence determinants are clearly higher than those with non-sequence determinants. (TIF) [file pcbi.1006451.s005.tif]

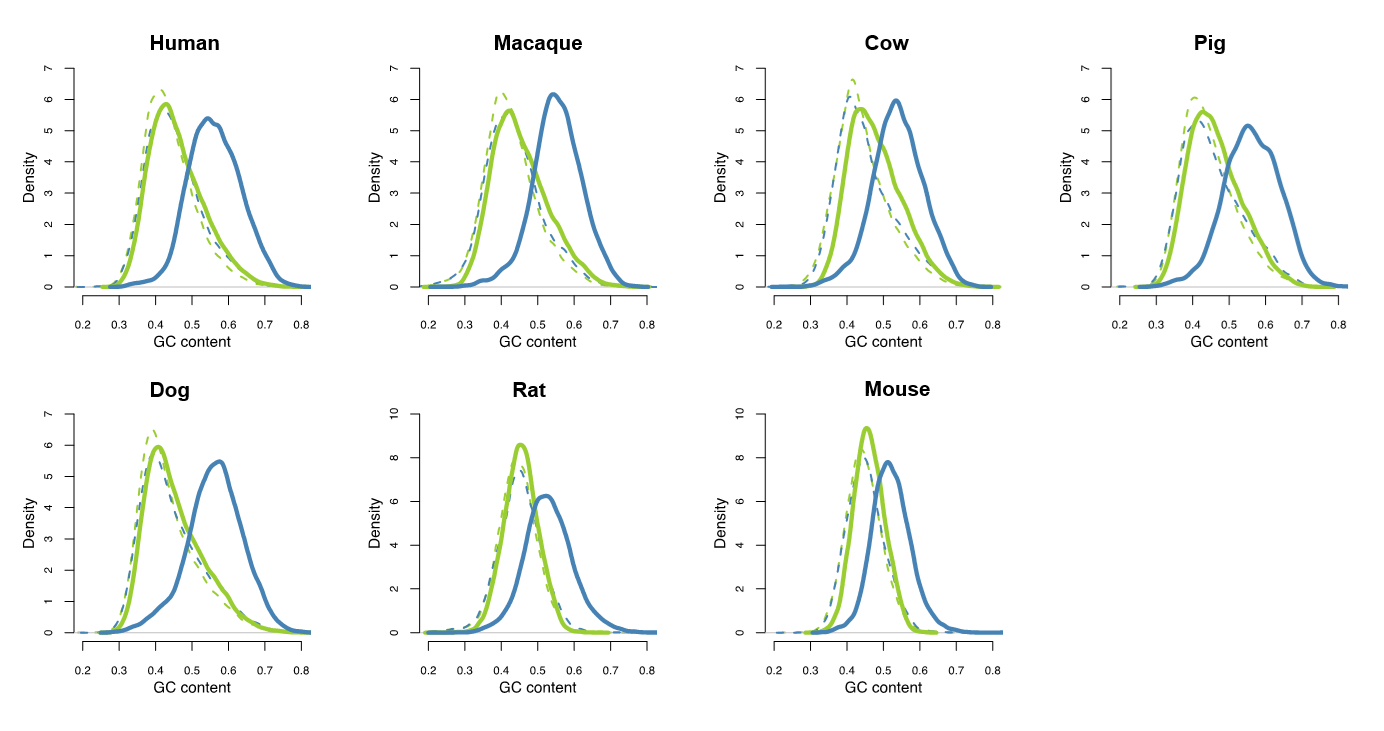

Supplement: S7 Fig — (TIF) [file pcbi.1006451.s007.tif]
