# Supplementary material for: Functional conservation of sequence determinants at rapidly evolving regulatory regions across mammals
Source: PLoS Comput Biol. 2018 Oct 5;14(10):e1006451. doi: 10.1371/journal.pcbi.1006451 (PMC6192654; doi:10.1371/journal.pcbi.1006451)
Supplement: S1 Table — (PDF) [file pcbi.1006451.s008.pdf]

	Enhancer	Promoter
Human	0.088	0.026
Macaque	0.054	0.019
Cow	0.061	0.032
Pig	0.075	0.021
Dog	0.062	0.030
Rat	0.065	0.029
Mouse	0.057	0.045
Mean	0.066	0.029
