# Supplementary material for: Functional conservation of sequence determinants at rapidly evolving regulatory regions across mammals
Source: PLoS Comput Biol. 2018 Oct 5;14(10):e1006451. doi: 10.1371/journal.pcbi.1006451 (PMC6192654; doi:10.1371/journal.pcbi.1006451)
Supplement: S2 Table — Enhancer sequence determinants are generally concentrated in the low OR ranges. (PDF) [file pcbi.1006451.s009.pdf]

FDR ≤0.05	OR criteria	Number of sequence determinants										
		6mer	7mer	8mer	9mer	10mer	11mer	12mer	13mer	14mer	15mer	Total
Human	1.0~1.2	1540	4647	8809	6831	226	28	16	9	8	6	22120
	1.2~1.4	416	2386	9053	19166	12918	1681	190	82	45	24	45961
	1.4~1.6	0	80	1628	6127	14057	6980	414	71	52	29	29438
	1.6~1.8	0	1	78	1355	2843	3151	196	20	12	4	7660
	1.8~2.0	0	0	15	228	434	854	67	5	0	1	1604
	≥2.0	0	0	2	45	108	300	38	5	3	3	504
Macaque	1.0~1.2	1388	3601	5680	1119	29	7	6	6	6	5	11847
	1.2~1.4	600	2825	7780	13152	3077	210	39	14	6	5	27708
	1.4~1.6	108	772	3539	5797	9086	1286	75	23	11	7	20704
	1.6~1.8	2	77	846	1928	3480	699	29	10	0	1	7072
	1.8~2.0	0	4	119	620	778	272	13	3	0	0	1809
	≥2.0	0	0	25	195	236	129	15	4	6	5	615
Cow	1.0~1.2	1091	3074	5742	2861	178	47	29	23	23	22	13090
	1.2~1.4	786	3392	11314	22703	11555	1082	96	51	31	20	51030
	1.4~1.6	84	758	4926	14669	25182	4917	269	90	47	27	50969
	1.6~1.8	0	16	567	4104	10237	3515	198	40	15	14	18706
	1.8~2.0	0	0	35	753	2709	1720	112	17	5	5	5356
	≥2.0	0	0	3	181	700	985	66	20	7	6	1968
Pig	1.0~1.2	1696	4842	7754	3227	61	17	8	6	5	5	17621
	1.2~1.4	139	1238	6590	13919	5484	584	113	38	21	12	28138
	1.4~1.6	0	1	371	2881	7128	2778	198	63	36	13	13469
	1.6~1.8	0	0	17	468	1677	1238	93	12	8	10	3523
	1.8~2.0	0	0	1	63	242	310	20	5	4	2	647
	≥2.0	0	0	0	12	48	98	5	0	2	0	165
Dog	1.0~1.2	1857	4637	6176	1505	45	28	17	14	12	13	14304
	1.2~1.4	40	704	3436	7423	1654	230	88	40	27	21	13663
	1.4~1.6	0	2	353	1111	2825	676	92	49	25	15	5148
	1.6~1.8	0	0	32	197	1008	322	29	12	11	8	1619
	1.8~2.0	0	0	1	24	143	84	5	2	0	3	262
	≥2.0	0	0	1	6	38	22	9	5	5	5	91
Rat	1.0~1.2	1438	3467	5061	1231	120	109	101	79	55	48	11709
	1.2~1.4	2	88	1114	4710	991	244	163	150	146	136	7744
	1.4~1.6	0	0	74	467	1992	489	158	118	106	92	3496
	1.6~1.8	0	0	4	83	790	227	45	30	39	42	1260
	1.8~2.0	0	0	0	18	120	52	12	4	11	13	230

	≥2.0	0	0	0	1	22	29	10	4	0	2	68
Mouse	1.0~1.2	1302	3293	5217	889	49	42	33	19	15	11	10870
	1.2~1.4	12	320	2494	8092	1457	152	63	46	43	29	12708
	1.4~1.6	0	2	167	1307	4388	361	101	63	49	43	6481
	1.6~1.8	0	0	16	119	1431	177	18	10	13	14	1798
	1.8~2.0	0	0	2	15	270	63	5	3	1	3	362
	≥2.0	0	0	0	5	87	47	18	2	0	0	159
