# Supplementary material for: Functional conservation of sequence determinants at rapidly evolving regulatory regions across mammals
Source: PLoS Comput Biol. 2018 Oct 5;14(10):e1006451. doi: 10.1371/journal.pcbi.1006451 (PMC6192654; doi:10.1371/journal.pcbi.1006451)
Supplement: S3 Table — Promoter sequence determinants are generally concentrated in the high OR ranges. (PDF) [file pcbi.1006451.s010.pdf]

FDR ≤0.05	OR	Number of sequence determinants										
		6mer	7mer	8mer	9mer	10mer	11mer	12mer	13mer	14mer	15mer	Total
Human	1.0~1.2	221	665	1112	167	7	3	1	1	2	5	2184
	1.2~1.4	198	718	2276	3837	1010	62	16	22	28	24	8191
	1.4~1.6	132	572	1925	3777	2462	153	25	12	6	7	9071
	1.6~1.8	120	497	1553	2306	1991	180	12	5	1	0	6665
	1.8~2.0	109	459	1441	1367	1413	222	17	2	1	0	5031
	≥2.0	924	4179	16231	24123	17007	5897	1467	418	162	75	70483
Macaque	1.0~1.2	197	659	764	43	0	0	0	0	0	0	1663
	1.2~1.4	209	711	2371	2670	389	17	3	1	6	7	6384
	1.4~1.6	137	622	1749	3753	1217	42	3	3	1	0	7527
	1.6~1.8	141	509	1370	2308	1274	67	2	0	0	0	5671
	1.8~2.0	95	448	1182	1463	1028	73	6	2	2	3	4302
	≥2.0	950	4293	15570	20048	12086	3056	575	163	57	30	56828
Cow	1.0~1.2	278	858	1324	309	12	5	6	9	8	6	2815
	1.2~1.4	211	879	2814	4551	1623	101	15	18	18	18	10248
	1.4~1.6	196	692	2401	3860	3185	300	19	12	15	11	10691
	1.6~1.8	136	605	1964	2105	1821	316	23	14	12	8	7004
	1.8~2.0	124	520	1814	1483	1062	270	18	8	6	4	5309
	≥2.0	752	3416	14049	23049	14514	4759	1209	372	112	61	62293
Pig	1.0~1.2	304	722	803	35	0	0	0	0	0	0	1864
	1.2~1.4	236	1039	2713	2795	494	14	2	0	0	0	7293
	1.4~1.6	176	781	2242	3742	1614	59	13	3	1	0	8631
	1.6~1.8	156	630	1579	1972	1348	101	22	3	1	0	5812
	1.8~2.0	114	472	1340	1210	923	124	22	8	5	3	4221
	≥2.0	713	3278	12489	16898	9956	3178	770	260	101	57	47700
Dog	1.0~1.2	208	485	698	125	15	10	10	6	4	3	1564
	1.2~1.4	148	612	1495	2376	820	72	32	38	32	29	5654
	1.4~1.6	117	556	1401	2041	1682	188	35	27	40	42	6129
	1.6~1.8	112	524	1342	1258	1229	227	32	18	12	10	4764
	1.8~2.0	121	497	1384	869	840	226	47	20	16	13	4033
	≥2.0	876	3798	14324	20708	16319	7597	2664	1046	529	339	68200
Rat	1.0~1.2	344	899	1320	160	8	1	0	1	1	0	2734
	1.2~1.4	212	881	2528	3705	728	52	10	7	3	2	8128
	1.4~1.6	177	698	2369	2953	2084	148	20	9	5	3	8466
	1.6~1.8	145	579	1966	1528	1253	121	27	12	8	7	5646
	1.8~2.0	98	503	1766	956	729	121	36	10	5	2	4226

	≥2.0	754	3335	13505	19944	7896	1485	352	105	35	19	47430
Mouse	1.0~1.2	355	963	1912	559	17	8	5	5	6	3	3833
	1.2~1.4	196	806	2652	5227	1686	148	52	29	25	20	10841
	1.4~1.6	152	712	2432	3175	3814	352	87	45	32	20	10821
	1.6~1.8	145	622	2304	1764	1805	296	94	58	30	16	7134
	1.8~2.0	126	563	2160	1380	879	201	78	34	24	21	5466
	≥2.0	759	3328	13831	24021	11190	2594	664	248	128	71	56834
