# Supplementary material for: Functional conservation of sequence determinants at rapidly evolving regulatory regions across mammals
Source: PLoS Comput Biol. 2018 Oct 5;14(10):e1006451. doi: 10.1371/journal.pcbi.1006451 (PMC6192654; doi:10.1371/journal.pcbi.1006451)
Supplement: S4 Table — Numbers of intersecting significant species sequence determinants from all seven species are shown in parentheses. Note that the ORs in this table represent the minimum ORs among the seven species. (PDF) [file pcbi.1006451.s011.pdf]

FDR≤0.05	OR	Sequence Determinant Length										
		6mer	7mer	8mer	9mer	10mer	11mer	12mer	13mer	14mer	15mer	Total
Enhancer	1.0~1.1	992 (559)	3383 (928)	9963 (442)	18943 (9)	16541 (0)	616 (0)	28 (0)	1 (0)	7 (0)	4 (0)	50478 (1938)
	1.1~1.2	81 (81)	491 (453)	2320 (1533)	5519 (534)	7257 (10)	275 (5)	10 (0)	1 (0)	0 (0)	0 (0)	15954 (2616)
	1.2~1.3	0 (0)	13 (13)	161 (141)	7579 (549)	1669 (57)	94 (6)	13 (8)	0 (4)	0 (0)	0 (0)	2707 (778)
	≥1.3	0 (0)	0 (0)	4 (3)	63 (58)	256 (83)	29 (12)	4 (2)	8 (1)	0 (0)	0 (0)	364 (160)
Promoter	1.0~1.1	149 (87)	52 (133)	1594 (47)	2940 (2)	814 (0)	29 (0)	2 (0)	0 (0)	2 (0)	1 (0)	5583 (269)
	1.1~1.2	105 (105)	451 (341)	1204 (590)	1914 (219)	801 (12)	39 (0)	2 (0)	2 (1)	0 (0)	1 (1)	4519 (1269)
	1.2~1.3	111 (111)	438 (422)	1049 (729)	1284 (768)	667 (179)	55 (15)	2 (1)	0 (0)	0 (0)	0 (0)	3606 (2225)
	≥1.3	1023 (1023)	4330 (4330)	14245 (14179)	16440 (16337)	7748 (7654)	1452 (1448)	305 (305)	81 (81)	24 (24)	17 (17)	45665 (45439)
