# Supplementary material for: Functional conservation of sequence determinants at rapidly evolving regulatory regions across mammals
Source: PLoS Comput Biol. 2018 Oct 5;14(10):e1006451. doi: 10.1371/journal.pcbi.1006451 (PMC6192654; doi:10.1371/journal.pcbi.1006451)
Supplement: S5 Table — There are few enhancer sequence determinants with large effect size that are common in the 7 mammalian species. On the other hand, the proportion of overlapping promoter sequence determinants is consistent across different effect sizes. (PDF) [file pcbi.1006451.s012.pdf]

	OR	Human	Macaque	Cow	Pig	Dog	Rat	Mouse
Enhancer (all determinants)	≥1.0	0.39	0.50	0.37	0.48	0.56	0.55	0.63
	≥1.1	0.14	0.19	0.12	0.19	0.25	0.32	0.29
	≥1.2	0.032	0.042	0.023	0.049	0.084	0.12	0.087
	≥1.3	0.0056	0.0071	0.0033	0.011	0.022	0.031	0.020
Promoter (all determinants)	≥1.0	0.57	0.70	0.58	0.74	0.60	0.72	0.59
	≥1.1	0.53	0.65	0.54	0.70	0.58	0.68	0.56
	≥1.2	0.50	0.61	0.51	0.66	0.55	0.66	0.54
	≥1.3	0.48	0.58	0.50	0.64	0.53	0.65	0.53
Enhancer (10k)	≥1.0	0.39	0.49	0.37	0.48	0.56	0.55	0.62
Promoter (10k)	≥1.0	0.57	0.70	0.58	0.74	0.61	0.72	0.60
