# Supplementary material for: Functional conservation of sequence determinants at rapidly evolving regulatory regions across mammals
Source: PLoS Comput Biol. 2018 Oct 5;14(10):e1006451. doi: 10.1371/journal.pcbi.1006451 (PMC6192654; doi:10.1371/journal.pcbi.1006451)
Supplement: S6 Table — The numbers outside the parentheses are from LASSO with species sequence determinants, while those in the parentheses are from LASSO with common sequence determinants. (PDF) [file pcbi.1006451.s013.pdf]

FDR ≤0.05	OR	Number of sequence determinants										
		6mer	7mer	8mer	9mer	10mer	11mer	12mer	13mer	14mer	15mer	Total
Human	1.0~1.2	82 (56)	191 (116)	324 (181)	228 (212)	3 (85)	1 (2)	0 (0)	0 (0)	0 (0)	0 (0)	829 (652)
	1.2~1.4	16 (28)	83 (58)	287 (165)	681 (252)	408 (118)	56 (6)	5 (0)	0 (0)	1 (0)	1 (0)	1538 (627)
	1.4~1.6	0 (0)	6 (0)	109 (36)	277 (84)	721 (149)	231 (6)	12 (0)	0 (0)	1 (0)	0 (0)	1357 (275)
	1.6~1.8	0 (0)	0 (0)	3 (6)	89 (8)	189 (46)	156 (4)	9 (1)	0 (0)	0 (0)	0 (0)	446 (65)
	1.8~2.0	0 (0)	0 (0)	2 (0)	20 (1)	29 (4)	49 (0)	8 (0)	1 (0)	0 (0)	0 (0)	109 (5)
	≥2.0	0 (0)	0 (0)	0 (0)	7 (1)	7 (1)	24 (0)	3 (0)	0 (0)	1 (0)	0 (0)	42 (2)
Macaque	1.0~1.2	95 (45)	193 (84)	270 (133)	31 (135)	1 (79)	1 (1)	1 (0)	0 (0)	0 (0)	0 (0)	592 (477)
	1.2~1.4	28 (31)	118 (71)	293 (115)	562 (255)	106 (93)	8 (3)	0 (0)	1 (0)	1 (0)	0 (0)	1117 (568)
	1.4~1.6	6 (2)	50 (20)	161 (72)	344 (124)	460 (147)	42 (7)	1 (0)	1 (0)	0 (0)	0 (0)	1065 (372)
	1.6~1.8	0 (0)	3 (0)	71 (21)	110 (23)	269 (107)	37 (4)	0 (0)	1 (1)	0 (0)	0 (0)	491 (156)
	1.8~2.0	0 (0)	1 (0)	12 (4)	43 (10)	77 (28)	19 (3)	0 (0)	0 (0)	0 (0)	0 (0)	152 (45)
	≥2.0	0 (0)	0 (0)	2 (1)	18 (1)	29 (12)	17 (0)	0 (0)	0 (0)	0 (0)	0 (0)	66 (14)
Cow	1.0~1.2	33 (41)	99 (90)	169 (142)	49 (141)	8 (76)	0 (5)	1 (0)	0 (0)	2 (0)	1 (0)	362 (495)
	1.2~1.4	24 (35)	80 (92)	226 (191)	471 (302)	158 (133)	17 (3)	2 (0)	0 (0)	2 (0)	0 (0)	980 (756)
	1.4~1.6	5 (5)	26 (27)	122 (115)	323 (237)	468 (143)	58 (8)	3 (0)	1 (0)	0 (0)	1 (0)	1007 (535)
	1.6~1.8	0 (0)	1 (0)	26 (33)	114 (70)	303 (136)	55 (6)	2 (1)	1 (0)	0 (0)	0 (0)	502 (246)
	1.8~2.0	0 (0)	0 (0)	2 (2)	30 (9)	121 (68)	36 (3)	1 (0)	1 (0)	0 (0)	0 (0)	191 (82)
	≥2.0	0 (0)	0 (0)	0 (0)	12 (1)	36 (11)	44 (2)	3 (0)	0 (0)	0 (0)	0 (0)	95 (14)
Pig	1.0~1.2	109 (59)	253 (101)	379 (179)	120 (193)	2 (83)	2 (2)	0 (0)	0 (0)	0 (0)	0 (0)	865 (617)
	1.2~1.4	7 (5)	48 (38)	264 (138)	715 (274)	206 (180)	27 (6)	4 (0)	2 (0)	0 (0)	0 (0)	1273 (641)
	1.4~1.6	0 (0)	0 (0)	38 (21)	260 (61)	637 (158)	175 (5)	13 (1)	3 (0)	2 (0)	0 (0)	1128 (246)
	1.6~1.8	0 (0)	0 (0)	5 (1)	73 (3)	233 (29)	124 (1)	6 (1)	0 (0)	2 (0)	0 (0)	443 (35)
	1.8~2.0	0 (0)	0 (0)	0 (0)	12 (0)	29 (4)	33 (0)	3 (0)	1 (0)	0 (0)	0 (0)	78 (4)
	≥2.0	0 (0)	0 (0)	0 (0)	3 (0)	5 (0)	15 (0)	2 (0)	0 (0)	0 (0)	0 (0)	25 (0)
Dog	1.0~1.2	197 (51)	412 (104)	455 (151)	106 (130)	4 (44)	4 (1)	2 (0)	1 (0)	1 (0)	1 (0)	1183 (481)
	1.2~1.4	5 (4)	83 (25)	400 (120)	856 (232)	146 (124)	24 (1)	6 (0)	2 (0)	5 (0)	1 (0)	1528 (506)
	1.4~1.6	0 (0)	1 (0)	67 (7)	215 (43)	517 (113)	86 (2)	9 (1)	9 (0)	2 (0)	1 (0)	907 (166)
	1.6~1.8	0 (0)	0 (0)	5 (1)	33 (2)	249 (34)	61 (0)	2 (0)	2 (0)	0 (0)	0 (0)	352 (37)
	1.8~2.0	0 (0)	0 (0)	0 (0)	9 (0)	36 (9)	16 (0)	0 (0)	1 (0)	0 (0)	1 (0)	63 (9)
	≥2.0	0 (0)	0 (0)	0 (0)	3 (0)	16 (1)	5 (0)	1 (0)	0 (0)	0 (0)	0 (0)	25 (1)
Rat	1.0~1.2	207 (64)	470 (120)	642 (175)	156 (151)	12 (39)	4 (3)	3 (1)	4 (0)	4 (0)	4 (0)	1506 (553)
	1.2~1.4	0 (0)	22 (5)	270 (104)	1018 (278)	212 (189)	39 (13)	9 (0)	10 (0)	7 (0)	5 (0)	1592 (589)
	1.4~1.6	0 (0)	0 (0)	22 (3)	159 (36)	623 (114)	120 (10)	27 (1)	12 (0)	11 (0)	8 (0)	982 (164)
	1.6~1.8	0 (0)	0 (0)	3 (0)	30 (1)	262 (20)	71 (5)	9 (0)	2 (0)	2 (0)	5 (0)	384 (26)
	1.8~2.0	0 (0)	0 (0)	0 (0)	7 (0)	36 (7)	20 (0)	1 (0)	0 (0)	3 (0)	1 (0)	68 (7)
	≥2.0	0 (0)	0 (0)	0 (0)	0 (0)	8 (0)	7 (0)	2 (0)	0 (0)	0 (0)	0 (0)	17 (0)
Mouse	1.0~1.2	171 (66)	327 (122)	472 (194)	77 (169)	3 (65)	2 (3)	4 (0)	0 (0)	2 (0)	0 (0)	1058 (619)
	1.2~1.4	3 (0)	45 (30)	343 (159)	1028 (369)	155 (200)	23 (9)	8 (1)	3 (0)	5 (0)	2 (0)	1615 (768)
	1.4~1.6	0 (0)	1 (0)	38 (10)	295 (79)	804 (210)	65 (8)	16 (1)	6 (0)	3 (0)	4 (0)	1232 (308)

	1.6~1.8	0 (0)	0 (0)	6 (0)	35 (12)	322 (82)	36 (4)	2 (0)	0 (0)	1 (0)	0 (0)	402 (98)
	1.8~2.0	0 (0)	0 (0)	2 (1)	2 (2)	65 (11)	12 (0)	2 (0)	0 (0)	0 (0)	0 (0)	83 (14)
	≥2.0	0 (0)	0 (0)	0 (0)	2 (1)	23 (5)	6 (1)	2 (0)	0 (0)	0 (0)	0 (0)	33 (7)
