# Supplementary material for: Functional conservation of sequence determinants at rapidly evolving regulatory regions across mammals
Source: PLoS Comput Biol. 2018 Oct 5;14(10):e1006451. doi: 10.1371/journal.pcbi.1006451 (PMC6192654; doi:10.1371/journal.pcbi.1006451)
Supplement: S7 Table — The numbers outside the parentheses are from LASSO with species sequence determinants, while those within the parentheses are from LASSO with common sequence determinants. (PDF) [file pcbi.1006451.s014.pdf]

FDR ≤0.05	OR	Number of sequence determinants										
		6mer	7mer	8mer	9mer	10mer	11mer	12mer	13mer	14mer	15mer	Total
Human	1.0~1.2	12 (4)	19 (11)	10 (15)	2 (21)	0 (0)	0 (0)	0 (0)	0 (0)	0 (0)	0 (0)	43 (51)
	1.2~1.4	7 (9)	20 (19)	37 (16)	46 (21)	10 (4)	0 (0)	1 (0)	0 (0)	1 (0)	1 (0)	123 (69)
	1.4~1.6	4 (6)	11 (18)	26 (22)	44 (19)	19 (9)	2 (1)	0 (0)	0 (0)	0 (0)	0 (0)	106 (75)
	1.6~1.8	8 (10)	9 (18)	20 (16)	34 (20)	19 (11)	0 (0)	0 (0)	0 (0)	0 (0)	0 (0)	90 (75)
	1.8~2.0	6 (7)	11 (12)	14 (15)	20 (17)	14 (6)	3 (1)	0 (0)	0 (0)	0 (0)	0 (0)	68 (58)
	≥2.0	64 (95)	177 (225)	300 (284)	219 (183)	110 (64)	33 (12)	8 (2)	1 (0)	0 (0)	0 (0)	912 (865)
Macaque	1.0~1.2	8 (6)	7 (3)	5 (7)	0 (11)	0 (0)	0 (0)	0 (0)	0 (0)	0 (0)	0 (0)	20 (27)
	1.2~1.4	3 (3)	15 (12)	26 (11)	16 (16)	1 (6)	0 (0)	0 (0)	0 (0)	0 (0)	0 (0)	61 (48)
	1.4~1.6	6 (8)	6 (10)	10 (10)	27 (17)	4 (3)	0 (0)	0 (0)	0 (0)	0 (0)	0 (0)	53 (48)
	1.6~1.8	10 (4)	10 (4)	10 (12)	13 (18)	10 (7)	0 (0)	0 (0)	0 (0)	0 (0)	0 (0)	53 (45)
	1.8~2.0	5 (3)	10 (13)	8 (6)	10 (18)	9 (3)	0 (0)	0 (0)	0 (0)	0 (0)	0 (0)	42 (43)
	≥2.0	90 (100)	158 (183)	182 (211)	106 (120)	47 (40)	9 (9)	0 (1)	0 (0)	0 (0)	0 (0)	592 (664)
Cow	1.0~1.2	12 (5)	17 (17)	23 (32)	4 (33)	0 (13)	0 (0)	0 (0)	0 (0)	0 (0)	0 (0)	56 (100)
	1.2~1.4	7 (10)	27 (14)	35 (40)	57 (28)	8 (9)	0 (0)	0 (0)	1 (0)	1 (0)	1 (0)	137 (101)
	1.4~1.6	14 (14)	11 (8)	43 (34)	35 (43)	23 (13)	3 (0)	0 (0)	0 (0)	1 (0)	0 (0)	130 (112)
	1.6~1.8	4 (9)	22 (26)	25 (21)	21 (27)	13 (9)	2 (0)	0 (0)	0 (0)	0 (0)	1 (0)	88 (92)
	1.8~2.0	5 (10)	15 (21)	22 (19)	20 (27)	15 (9)	1 (0)	0 (0)	0 (0)	0 (0)	0 (0)	78 (86)
	≥2.0	58 (77)	143 (162)	245 (292)	213 (186)	88 (67)	30 (13)	3 (4)	1 (1)	1 (0)	0 (0)	782 (802)
Pig	1.0~1.2	20 (9)	20 (20)	28 (29)	0 (37)	0 (8)	0 (0)	0 (0)	0 (0)	0 (0)	0 (0)	68 (103)
	1.2~1.4	8 (8)	25 (17)	56 (33)	40 (43)	7 (6)	0 (0)	0 (0)	0 (0)	0 (0)	0 (0)	136 (107)
	1.4~1.6	12 (10)	17 (12)	42 (36)	82 (35)	17 (11)	1 (0)	0 (0)	0 (0)	0 (0)	0 (0)	171 (104)
	1.6~1.8	9 (9)	12 (23)	28 (38)	44 (28)	18 (11)	0 (1)	1 (0)	0 (0)	0 (0)	0 (0)	112 (110)
	1.8~2.0	6 (5)	18 (15)	20 (22)	20 (21)	12 (7)	1 (0)	1 (0)	0 (0)	0 (0)	0 (0)	78 (70)
	≥2.0	67 (72)	156 (165)	281 (278)	180 (176)	85 (63)	19 (18)	1 (2)	2 (0)	0 (0)	1 (0)	792 (774)
Dog	1.0~1.2	6 (6)	11 (16)	12 (17)	1 (26)	0 (9)	0 (1)	0 (0)	1 (0)	0 (0)	0 (0)	31 (75)
	1.2~1.4	8 (6)	13 (3)	20 (25)	35 (28)	8 (9)	0 (0)	1 (0)	0 (0)	0 (0)	0 (0)	85 (71)
	1.4~1.6	2 (8)	12 (9)	11 (16)	28 (15)	15 (8)	2 (3)	1 (0)	0 (0)	2 (0)	2 (0)	75 (59)
	1.6~1.8	3 (3)	10 (11)	18 (5)	27 (17)	10 (3)	1 (0)	0 (0)	1 (0)	1 (0)	0 (0)	71 (39)
	1.8~2.0	11 (6)	12 (12)	19 (12)	13 (9)	5 (4)	6 (0)	0 (0)	1 (0)	0 (0)	0 (0)	67 (43)
	≥2.0	62 (88)	141 (148)	277 (255)	191 (156)	125 (57)	35 (6)	18 (0)	7 (0)	3 (0)	2 (0)	861 (710)
Rat	1.0~1.2	20 (8)	45 (20)	31 (32)	5 (40)	0 (17)	0 (0)	0 (0)	0 (0)	0 (0)	0 (0)	101 (117)
	1.2~1.4	15 (5)	29 (23)	56 (52)	95 (62)	13 (16)	1 (2)	0 (0)	0 (0)	0 (0)	0 (0)	209 (160)
	1.4~1.6	7 (13)	26 (20)	52 (27)	74 (44)	41 (14)	2 (0)	0 (0)	0 (0)	0 (0)	0 (0)	202 (118)
	1.6~1.8	8 (9)	23 (27)	53 (36)	50 (28)	21 (17)	0 (3)	0 (0)	0 (0)	0 (0)	0 (0)	155 (120)
	1.8~2.0	5 (10)	16 (26)	52 (38)	15 (15)	23 (8)	2 (0)	0 (1)	0 (0)	0 (0)	0 (0)	113 (98)
	≥2.0	83 (92)	180 (191)	359 (349)	293 (210)	81 (74)	14 (14)	2 (3)	0 (1)	0 (0)	0 (0)	1012 (934)
Mouse	1.0~1.2	11 (4)	27 (16)	46 (15)	14 (10)	0 (1)	0 (0)	1 (0)	0 (0)	0 (0)	0 (0)	99 (46)
	1.2~1.4	6 (4)	15 (16)	48 (22)	88 (30)	21 (3)	0 (0)	0 (0)	0 (0)	1 (0)	0 (0)	179 (75)
	1.4~1.6	1 (7)	21 (19)	36 (14)	60 (18)	61 (8)	4 (0)	0 (0)	0 (0)	0 (0)	1 (0)	184 (66)

	1.6~1.8	6 (6)	16 (7)	42 (11)	20 (10)	40 (2)	5 (3)	0 (0)	2 (0)	0 (0)	0 (0)	131 (39)
	1.8~2.0	6 (11)	21 (12)	25 (13)	16 (5)	21 (6)	5 (0)	1 (1)	3 (0)	0 (0)	0 (0)	98 (48)
	≥2.0	62 (81)	152 (145)	302 (158)	293 (108)	87 (15)	15 (5)	9 (0)	0 (1)	2 (0)	2 (0)	924 (513)
