# Supplementary material for: Functional conservation of sequence determinants at rapidly evolving regulatory regions across mammals
Source: PLoS Comput Biol. 2018 Oct 5;14(10):e1006451. doi: 10.1371/journal.pcbi.1006451 (PMC6192654; doi:10.1371/journal.pcbi.1006451)
Supplement: S8 Table — The model used is: log2OR ~ GC contents + TFBS Frequency + GC contents × TFBS Frequency + ε. NS indicates that the interaction terms were not statistically significant at P = 0.05. In such cases, we conducted log2OR ~ GC contents + TFBS Frequency + GC contents + ε model instead. R2 values of the models are also provided. (PDF) [file pcbi.1006451.s015.pdf]

Dataset	Region	Variable	Estimate	Standard error	P-value	SSR
Macaque	Enhancer (n=3483, R^2=0.171)	GC contents	0.68	0.026	$< 1\times10^{-15}$	0.167
		TFBS frequency	0.80	0.12	7.32×10^{-12}	0.011
		GC × TFBS	NS			
	Promoter (n=821, R^2=0.381)	GC contents	6.09	0.39	$< 1\times10^{-15}$	0.181
		TFBS frequency	23.09	4.45	2.61×10^{-7}	0.020
		GC × TFBS	-45.16	7.05	2.49×10^{-10}	0.031
Cow	Enhancer (n=3137, R^2=0.204)	GC contents	0.74	0.06	$< 1\times10^{-15}$	0.041
		TFBS frequency	-0.01	0.51	0.98	0.000
		GC × TFBS	1.80	0.89	0.04	0.01
	Promoter (n=1271, R^2=0.364)	GC contents	5.11	0.27	$< 1\times10^{-15}$	0.186
		TFBS frequency	20.32	2.53	1.99×10^{-15}	0.033
		GC × TFBS	-41.45	3.90	$< 1\times10^{-15}$	0.057
Pig	Enhancer (n=3812, R^2=0.071)	GC contents	0.44	0.03	$< 1\times10^{-15}$	0.071
		TFBS frequency	0.61	0.11	2.81×10^{-08}	0.008
		GC × TFBS	NS			
	Promoter (n=1357, R^2=0.330)	GC contents	5.28	0.28	$< 1\times10^{-15}$	0.179
		TFBS frequency	20.93	2.67	8.21×10^{-15}	0.031
		GC × TFBS	-41.24	4.25	$< 1\times10^{-15}$	0.047
Dog	Enhancer (n=4058, R^2=0.0267)	GC contents	0.25	0.02	$< 1\times10^{-15}$	0.025
		TFBS frequency	0.73	0.11	4.86×10^{-12}	0.015
		GC × TFBS	NS			
	Promoter (n=1190, R^2=0.384)	GC contents	5.85	0.29	$< 1\times10^{-15}$	0.213
		TFBS frequency	22.04	2.41	$< 1\times10^{-15}$	0.044
		GC × TFBS	-41.16	3.59	$< 1\times10^{-15}$	0.068
Rat	Enhancer (n=4549, R^2=0.0197)	GC contents	0.17	0.02	2.76×10^{-14}	0.013
		TFBS frequency	0.80	0.10	1.53×10^{-14}	0.013
		GC × TFBS	NS			
	Promoter (n=1792, R^2=0.387)	GC contents	6.00	0.24	$< 1\times10^{-15}$	0.206
		TFBS frequency	25.86	2.44	$< 1\times10^{-15}$	0.039
		GC × TFBS	-51.00	3.92	$< 1\times10^{-15}$	0.058
