# Supplementary material for: Functional conservation of sequence determinants at rapidly evolving regulatory regions across mammals
Source: PLoS Comput Biol. 2018 Oct 5;14(10):e1006451. doi: 10.1371/journal.pcbi.1006451 (PMC6192654; doi:10.1371/journal.pcbi.1006451)
Supplement: S9 Table — The data sets used in this analysis are from results of LASSO selected common sequence determinants. Test results whose interaction terms are NS indicate that the interaction terms were not statistically significant at P = 0.05, and we conducted log2OR ~ GC contents + TFBS Frequency + GC contents + ε model instead. R2 values of the models are also provided. (PDF) [file pcbi.1006451.s016.pdf]

Dataset	Region	Variable	Estimate	Standard error	P-value	SSR
Human	Enhancer (n=1626, R^2=0.0940)	GC contents	0.44	0.034	$<1\times10^{-15}$	0.0938
		TFBS Frequency	0.74	0.14	1.36×10^{-7}	0.0157
		GC × TFBS	NS			
	Promoter (n=1193, R^2=0.359)	GC contents	6.27	0.33	$<1\times10^{-15}$	0.194
		TFBS Frequency	29.91	3.54	$<1\times10^{-15}$	0.038
		GC × TFBS	-55.54	5.40	$<1\times10^{-15}$	0.057
Mouse	Enhancer (n=1814, R^2=0.0456)	GC contents	0.31	0.038	$<1\times10^{-15}$	0.0349
		TFBS Frequency	1.010	0.15	1.52×10^{-11}	0.0243
		GC × TFBS	NS			
	Promoter (n=787, R^2=0.286)	GC contents	4.41	0.36	$<1\times10^{-15}$	0.135
		TFBS Frequency	18.14	3.59	5.62×10^{-7}	0.023
		GC × TFBS	-34.50	5.53	7.23×10^{-10}	0.036
