# Supplementary material for: Functional conservation of sequence determinants at rapidly evolving regulatory regions across mammals
Source: PLoS Comput Biol. 2018 Oct 5;14(10):e1006451. doi: 10.1371/journal.pcbi.1006451 (PMC6192654; doi:10.1371/journal.pcbi.1006451)
Supplement: S10 Table — We used same models that were used in Table 3 and substituted -log10min(P) for TFBS frequency. (PDF) [file pcbi.1006451.s017.pdf]

Dataset	Region	Variable	Estimate	Standard error	P-value	SSR
Human	Enhancer (n=4321, R^2=0.1476)	GC contents	0.57	0.021	$<1\times10^{-15}$	0.143
		$-\log_{10}\min(P)$	0.045	0.0041	$<1\times10^{-15}$	0.024
	Promoter (n=1342, R^2=0.3204)	GC contents	7.88	0.72	$<1\times10^{-15}$	0.060
		$-\log_{10}\min(P)$	0.81	0.17	1.56×10^{-06}	0.012
		GC × $-\log_{10}\min(P)$	-1.16	0.25	2.88×10^{-06}	0.011
Mouse	Enhancer (n=4423, R^2=0.0458)	GC contents	0.24	0.025	$<1\times10^{-15}$	0.020
		$-\log_{10}\min(P)$	0.051	0.0041	$<1\times10^{-15}$	0.025
	Promoter (n=1615, R^2=0.3052)	GC contents	6.64	0.41	$<1\times10^{-15}$	0.106
		$-\log_{10}\min(P)$	0.64	0.092	2.93×10^{-12}	0.020
		GC × $-\log_{10}\min(P)$	-1.19	0.14	$<1\times10^{-15}$	0.030
