# Supplementary material for: Functional conservation of sequence determinants at rapidly evolving regulatory regions across mammals
Source: PLoS Comput Biol. 2018 Oct 5;14(10):e1006451. doi: 10.1371/journal.pcbi.1006451 (PMC6192654; doi:10.1371/journal.pcbi.1006451)
Supplement: S11 Table — The data sets used in this analysis are from results of exhaustive search. When interaction term of the model was not significant (NS), we conducted log2OR ~ GC contents + TFBS Frequency + ε model instead. (PDF) [file pcbi.1006451.s018.pdf]

Dataset	Region	Variable	Estimate	Standard error	P-value	SSR
Human	Enhancer (R^2=0.102)	GC contents	0.58	0.0074	$< 1\times10^{-15}$	0.051
		TFBS Frequency	1.45	0.061	$< 1\times10^{-15}$	0.005
		GC × TFBS	-1.51	0.11	$< 1\times10^{-15}$	0.002
	Promoter (R^2=0.526)	GC contents	8.45	0.032	$<1\times10^{-15}$	0.333
		TFBS Frequency	30.88	0.26	$<1\times10^{-15}$	0.067
		GC × TFBS	-54.54	0.42	$<1\times10^{-15}$	0.078
Mouse	Enhancer (R^2=0.0235)	GC contents	0.19	0.0095	$<1\times10^{-15}$	0.012
		TFBS Frequency	0.87	0.034	$<1\times10^{-15}$	0.020
		GC × TFBS	NS			
	Promoter (R^2=0.544)	GC contents	7.68	0.029	$<1\times10^{-15}$	0.347
		TFBS Frequency	30.41	0.23	$<1\times10^{-15}$	0.086
		GC × TFBS	-55.26	0.35	$<1\times10^{-15}$	0.117
Common	Enhancer (R^2=0.277)	GC contents	0.59	0.0062	$<1\times10^{-15}$	0.093
		TFBS Frequency	1.49	0.050	$<1\times10^{-15}$	0.009
		GC × TFBS	-1.47	0.086	$<1\times10^{-15}$	0.003
	Promoter (R^2=0.558)	GC contents	7.70	0.038	$<1\times10^{-15}$	0.303
		TFBS Frequency	33.71	0.36	$<1\times10^{-15}$	0.064
		GC × TFBS	-58.96	0.53	$<1\times10^{-15}$	0.0905
