# Supplementary material for: Functional conservation of sequence determinants at rapidly evolving regulatory regions across mammals
Source: PLoS Comput Biol. 2018 Oct 5;14(10):e1006451. doi: 10.1371/journal.pcbi.1006451 (PMC6192654; doi:10.1371/journal.pcbi.1006451)
Supplement: S12 Table — The 10,000 determinants were selected using stratified random sampling from the exhaustive search results. Columns refer to LASSO models trained for the seven species, and rows show test data sets to be predicted by the LASSO trained models. The values in parenthesis under the species names indicate the number of LASSO selected sequence determinants of enhancers (left) and promoters (right). AUC values out of parenthesis are receiver operating characteristic (ROC)-AUCs and those in parenthesis are precision-recall (PR)-AUCs. Note that the AUC values in diagonal terms are same-species prediction AUC and the other values in off-diagonal terms are inter-species prediction AUC values. (PDF) [file pcbi.1006451.s019.pdf]

		Trained LASSO model (Enhancer/Promoter)						
		Human (4320/1342)	Macaque (3482/821)	Cow (3137/1271)	Pig (3812/1357)	Dog (4058/1190)	Rat (4549/1792)	Mouse (4423/1615)
Test Data Sets (Enhancer)	Human	0.715 (0.687)	0.691 (0.658)	0.649 (0.627)	0.664 (0.638)	0.659 (0.635)	0.660 (0.637)	0.658 (0.634)
	Macaque	0.679 (0.666)	0.713 (0.696)	0.650 (0.639)	0.662 (0.650)	0.674 (0.659)	0.669 (0.657)	0.661 (0.649)
	Cow	0.651 (0.632)	0.662 (0.637)	0.714 (0.677)	0.658 (0.636)	0.657 (0.638)	0.652 (0.632)	0.660 (0.641)
	Pig	0.649 (0.650)	0.656 (0.652)	0.637 (0.637)	0.702 (0.698)	0.657 (0.655)	0.658 (0.658)	0.659 (0.659)
	Dog	0.627 (0.609)	0.644 (0.618)	0.614 (0.592)	0.642 (0.619)	0.717 (0.694)	0.648 (0.629)	0.639 (0.619)
	Rat	0.618 (0.607)	0.636 (0.617)	0.614 (0.602)	0.641 (0.624)	0.645 (0.630)	0.741 (0.725)	0.662 (0.643)
	Mouse	0.647 (0.631)	0.661 (0.632)	0.653 (0.629)	0.663 (0.640)	0.659 (0.636)	0.697 (0.669)	0.756 (0.731)
Test Data Sets (Promoter)	Human	0.966 (0.956)	0.967 (0.957)	0.963 (0.956)	0.957 (0.950)	0.959 (0.951)	0.960 (0.950)	0.958 (0.948)
	Macaque	0.968 (0.964)	0.967 (0.962)	0.963 (0.960)	0.955 (0.954)	0.959 (0.956)	0.961 (0.956)	0.960 (0.956)
	Cow	0.930 (0.923)	0.932 (0.923)	0.938 (0.930)	0.934 (0.928)	0.933 (0.919)	0.930 (0.919)	0.925 (0.914)
	Pig	0.934 (0.939)	0.936 (0.942)	0.938 (0.944)	0.946 (0.951)	0.931 (0.936)	0.933 (0.936)	0.928 (0.930)
	Dog	0.944 (0.935)	0.947 (0.937)	0.949 (0.942)	0.948 (0.942)	0.949 (0.931)	0.943 (0.931)	0.939 (0.925)
	Rat	0.899 (0.909)	0.906 (0.914)	0.904 (0.914)	0.895 (0.908)	0.896 (0.921)	0.916 (0.921)	0.909 (0.917)
	Mouse	0.917 (0.914)	0.921 (0.916)	0.918 (0.915)	0.916 (0.914)	0.914 (0.922)	0.929 (0.922)	0.929 (0.921)
