# Supplementary material for: Functional conservation of sequence determinants at rapidly evolving regulatory regions across mammals
Source: PLoS Comput Biol. 2018 Oct 5;14(10):e1006451. doi: 10.1371/journal.pcbi.1006451 (PMC6192654; doi:10.1371/journal.pcbi.1006451)
Supplement: S13 Table — The 10,000 determinants were selected using stratified random sampling from the exhaustive search results. Columns of the table are LASSO models that were trained from the seven species, and rows are test data sets to be predicted by the LASSO trained models. The values in parentheses under the species names are the number of LASSO selected sequence determinants of enhancers (left) and promoters (right). AUC values outside of parenthesis are receiver operating characteristic (ROC)-AUCs and those in parenthesis are precision-recall (PR)-AUCs. Note that the AUC values in diagonal terms are same-species prediction AUC and the other values in off-diagonal terms are inter-species prediction AUC values. (PDF) [file pcbi.1006451.s020.pdf]

		Trained LASSO model (Enhancer/Promoter)						
		Human (1626/1193)	Macaque (1632/875)	Cow (2128/1293)	Pig (1543/1268)	Dog (1200/997)	Rat (1339/1547)	Mouse (1814/787)
Test Data Sets (Enhancer)	Human	0.696 (0.668)	0.686 (0.656)	0.677 (0.645)	0.676 (0.651)	0.668 (0.639)	0.668 (0.641)	0.668 (0.641)
	Macaque	0.678 (0.667)	0.681 (0.666)	0.669 (0.654)	0.668 (0.659)	0.673 (0.657)	0.668 (0.654)	0.666 (0.654)
	Cow	0.694 (0.667)	0.694 (0.663)	0.716 (0.681)	0.694 (0.666)	0.687 (0.660)	0.695 (0.666)	0.701 (0.672)
	Pig	0.660 (0.661)	0.655 (0.653)	0.658 (0.655)	0.666 (0.663)	0.659 (0.656)	0.663 (0.661)	0.662 (0.659)
	Dog	0.638 (0.618)	0.647 (0.619)	0.635 (0.611)	0.644 (0.621)	0.645 (0.619)	0.642 (0.620)	0.638 (0.615)
	Rat	0.636 (0.627)	0.644 (0.629)	0.643 (0.631)	0.647 (0.637)	0.646 (0.634)	0.654 (0.637)	0.658 (0.642)
	Mouse	0.669 (0.651)	0.674 (0.650)	0.687 (0.662)	0.682 (0.662)	0.676 (0.652)	0.691 (0.667)	0.694 (0.667)
Test Data Sets (Promoter)	Human	0.967 (0.957)	0.967 (0.957)	0.959 (0.953)	0.960 (0.953)	0.960 (0.954)	0.959 (0.950)	0.960 (0.950)
	Macaque	0.969 (0.956)	0.967 (0.961)	0.960 (0.958)	0.960 (0.958)	0.961 (0.959)	0.962 (0.957)	0.962 (0.957)
	Cow	0.933 (0.923)	0.932 (0.922)	0.938 (0.930)	0.935 (0.928)	0.934 (0.927)	0.929 (0.916)	0.927 (0.914)
	Pig	0.939 (0.945)	0.938 (0.942)	0.942 (0.948)	0.947 (0.952)	0.941 (0.947)	0.934 (0.937)	0.933 (0.935)
	Dog	0.947 (0.939)	0.946 (0.936)	0.949 (0.943)	0.948 (0.941)	0.951 (0.943)	0.944 (0.932)	0.943 (0.930)
	Rat	0.904 (0.913)	0.905 (0.913)	0.901 (0.910)	0.898 (0.910)	0.903 (0.914)	0.915 (0.919)	0.913 (0.919)
	Mouse	0.920 (0.917)	0.921 (0.917)	0.917 (0.915)	0.916 (0.915)	0.917 (0.915)	0.928 (0.921)	0.927 (0.920)
