# Supplementary material for: Functional conservation of sequence determinants at rapidly evolving regulatory regions across mammals
Source: PLoS Comput Biol. 2018 Oct 5;14(10):e1006451. doi: 10.1371/journal.pcbi.1006451 (PMC6192654; doi:10.1371/journal.pcbi.1006451)
Supplement: S14 Table — Using the information described in Table 1, we conducted the same procedure of exhaustive search and LASSO prediction using 10K species sequence determinants. We then calculated AUCs and compared them to the original analysis results. (PDF) [file pcbi.1006451.s021.pdf]

		Train Data sets			
		Original analysis with whole region		Filtered analysis without conserved region	
		Human	Mouse	Human	Mouse
Test Data Sets (Enhancer)	Human	0.715 (4321)	0.658	0.701 (4125)	0.645
	Mouse	0.647	0.756 (4424)	0.638	0.746 (3728)
Test Data Sets (Promoter)	Human	0.966 (1343)	0.958	0.962 (1253)	0.945
	Mouse	0.917	0.929 (1616)	0.908	0.923 (1426)
